# Distinct neural mechanisms of alpha binaural beats and white noise for cognitive enhancement in young adults

**DOI:** 10.3934/Neuroscience.2025010

**Published:** 2025-05-20

**Authors:** Aini Ismafairus Abd Hamid, Nurfaten Hamzah, Siti Mariam Roslan, Nur Alia Amalin Suhardi, Muhammad Riddha Abdul Rahman, Faiz Mustafar, Hazim Omar, Asma Hayati Ahmad, Elza Azri Othman, Ahmad Nazlim Yusoff

**Affiliations:** 1 Department of Neurosciences, School of Medical Sciences, Health Campus, Universiti Sains Malaysia, Kubang Kerian, Kelantan, Malaysia; 2 Brain & Behaviour Cluster, School of Medical Sciences, Universiti Sains Malaysia, Kubang Kerian, Kelantan, Malaysia; 3 Hospital Pakar Universiti Sains Malaysia, Health Campus, Universiti Sains Malaysia, Kubang Kerian, Kelantan, Malaysia; 4 Faculty of Cognitive Sciences and Human Development, Universiti Malaysia Sarawak, Kota Samarahan, Sarawak, Malaysia; 5 School of Medical Imaging, Faculty of Health Sciences, Universiti Sultan Zainal Abidin, Kuala Nerus, Terengganu, Malaysia; 6 Department of Physiology, School of Medical Sciences, Health Campus, Universiti Sains Malaysia, Kubang Kerian, Kelantan, Malaysia; 7 Center for Diagnostic, Therapeutic and Investigative Studies, Faculty of Health Science, Universiti Kebangsaan Malaysia, Jalan Raja Muda Abdul Aziz, Wilayah Persekutuan Kuala Lumpur, Malaysia

**Keywords:** alpha binaural beats, white noise, functional connectivity, fMRI, cognitive enhancements, young adults

## Abstract

Young adulthood is a critical period marked by significant cognitive demands, requiring efficient brain function to manage academic, professional, and social challenges. Many young adults struggle with focus, stress management, and information processing. Emerging research suggests that auditory stimulation, specifically binaural beats and white noise, may enhance cognitive abilities and address these challenges. This exploratory study investigates the immediate effects of alpha binaural beats (ABB) and alpha binaural beats combined with white noise (AWN) on brain connectivity in young adults using functional magnetic resonance imaging (fMRI). Twenty-nine participants (n = 14 ABB, n = 15 AWN; mean age ≈ 22.14 years) were randomly assigned to receive either ABB or AWN during fMRI scans. Using dynamic independent component analysis (dyn-ICA), we examined the modulation of functional brain circuits during auditory stimulation. Preliminary findings revealed distinct and overlapping patterns of brain connectivity modulation of ABB and AWN. ABB primarily modulated connectivity within circuits involving frontoparietal, visual-motor, and multisensory regions, potentially enhancing cognitive flexibility, attentional control, and multisensory processing. Conversely, AWN primarily modulated connectivity in salience and default mode networks, with notable effects in limbic or reward regions, suggesting enhancements in focused attention and emotional processing. These preliminary results demonstrate that ABB and AWN differentially modulate brain networks on an immediate timescale. ABB may promote cognitive adaptability, while AWN enhances focused attention and emotional stability. Although behavioral effects were not assessed, these findings provide a neurobiological basis for understanding how these stimuli impact brain circuits. These preliminary findings may aid the development of personalized strategies for cognitive and emotional well-being. Given the exploratory nature, small sample size, and lack of concurrent behavioral data, these findings should be interpreted cautiously. Future research with rigorous designs, including control groups and behavioral measures, is needed to explore the long-term effects and applications of these interventions in various settings.

## 1. Introduction

Cognitive function, encompassing attention, memory, and executive functions [1]–[6], is crucial for young adults managing academic, professional, and social demands. During this developmental stage, individuals encounter increasing stressors related to academic performance, career planning, and complex social interactions [7]–[9], which place substantial demands on cognitive resources. Prolonged stress and cognitive overload can impair cognitive functioning [10],[11], disrupt sleep quality [12]–[14], and potentially impact long-term mental health. Therefore, proactive approaches to support health during young adulthood are essential for academic success, professional development, and overall well-being [15]–[17].

From a network neuroscience perspective, complex cognitive functions emerge from the dynamic interactions within and between large-scale functional brain networks. Recently, non-invasive auditory stimulation techniques, such as binaural beats, have received growing interest as potential modulators of the brain network activity [18]–[22]. Binaural beats are created by presenting two slightly different sound frequencies to each ear [20],[23]–[25], which produces the perception of a third tone that corresponds to the frequency difference between the two frequencies [20],[23]–[25]. Binaural beat stimulation within the alpha frequency range (7–11 Hz) [24],[25] has been hypothesized to influence brain oscillatory activity associated with relaxation [26], attention [27] and working memory [28].

Several studies suggest that alpha binaural beats (ABB) can improve cognitive performance [19],[25],[27] in attention, working memory, and emotional regulation. However, it is important to note that the scientific evidence supporting these effects remains inconclusive, with findings varying widely across different studies. Notably, Klichowski et al. [29] conducted a large-scale investigation involving 1000 participants and found no significant improvement in cognitive performance, as measured by Raven's Progressive Matrices and Matrix Reasoning Item Bank tests, following exposure to binaural beats. No brain imaging modalities were employed, and the study suggested potential adverse effects. Similarly, Orozco Perez et al. [30] reported a failure to enhance electroencephalogram (EEG) power and no significant changes in emotional arousal after binaural beat stimulation in the EEG study. Further caution is warranted based on systematic reviews such as that by Ingendoh et al. [25], who concluded that existing studies on binaural beat stimulation should be interpreted with caution, emphasizing the heterogeneity and inconsistency across psychophysiological and cognitive outcomes. Thus, while preliminary evidence points to potential benefits, the effects of binaural beats on brain function and cognitive performance remain controversial and require further systematic investigation.

Another auditory stimulus, white noise (WN), a broadband signal with uniform spectral density, has also been examined for its potential to influence cognitive processes [31]–[33]. White noise may enhance cognitive performance by improving signal detection and sensory gating, although its effects vary with factors such as age, baseline cognitive function, and task characteristics [31],[32],[34],[35]. Recent studies by Aloysius et al. [20] suggested that alpha-embedded binaural beats within WN (AWN) might enhance neuronal activation and information processing even further. This multimodal sound environment could be particularly beneficial for young adults, who frequently encounter noisy, distracting environments demanding high levels of cognitive control.

Despite the growing interest, several gaps remain. Previous studies have investigated ABB and AWN separately, with most relying on pre- and post-stimulation EEG design, which limits the spatial resolution and dynamic tracking of network-level changes. Moreover, relatively few studies have examined brain connectivity patterns during the stimulation period itself, particularly using high-resolution imaging modalities such as functional magnetic resonance imaging (fMRI).

Therefore, the present study employs fMRI combined with dynamic independent component analysis (dyn-ICA) to investigate how ABB and AWN may differentially modulate functional brain connectivity in young adults during binaural beat stimulation. This approach allows for a more spatially detailed examination of dynamic network interactions across cognitive, affective, and sensory domains.

This study is explicitly exploratory and preliminary in nature. Rather than testing a specific directional hypothesis, we aim to characterize neural modulation patterns associated with ABB and AWN stimulation. We acknowledge the exploratory aspect of this research, which is based on limited prior findings suggesting a potentially distinct effect [20],[36],[37]. We tentatively investigate whether AWN might induce distinct connectivity changes in critical brain areas associated with cognitive control, such as the dorsolateral prefrontal cortex and the orbitofrontal cortex [20],[36],[37] compared to ABB. Any observed patterns are interpreted with caution due to variability in previous findings and the preliminary nature of the current dataset. By positioning our work within the broader, ongoing discourse on auditory stimulation, we aim to provide preliminary insights that could guide future hypothesis-driven research and the development of non-invasive cognitive enhancement strategies for educational and clinical applications.

## 2. Materials and methods

### 2.1. Participants

Thirty-four healthy participants (mean age = 22.24 years, SD = 0.95) were recruited for this study and randomly assigned to the ABB or AWN experimental group. Due to technical complications, including improper protocol execution and file corruption during data acquisition, three participants from the ABB group and two from the AWN group were excluded, resulting in a final sample of 29 participants (n = 14 ABB, n = 15 AWN). A priori power analysis was conducted using G*Power 3.1.9.7 to estimate the sample size required to detect a medium effect size (Cohen's d = 0.5) using a two-tailed independent samples *t*-test with an alpha level of 0.05 and 80% power. The analysis indicated that 128 participants (64 per group) would be necessary for adequate statistical power. However, given the exploratory nature of this study, the final sample of 29 participants (14 in ABB; 15 in AWN) was considered appropriate for a preliminary investigation. This sample size aligns with methodological recommendations for pilot studies aimed at parameter estimation for future confirmatory trials [38]. Accordingly, the present study was explicitly framed as exploratory and hypothesis-generating.

Inclusion criteria required Malaysian citizenship, an age range of 18–35 years per the Youth Societies and Youth Development (Amendment) Act 2019 (Act A1602), and enrolment as third- or fourth-year undergraduate students in the School of Health Sciences, Universiti Sains Malaysia (USM). This population was selected due to their unique cognitive demands during this critical stage of their academic progression, which includes managing advanced coursework, practical placements, and preparing for future career requirements. Optimizing cognitive function during this period is essential for academic success, practical competency, and career readiness. Additionally, participants were required to achieve a working memory index score of 80 or above, as assessed by the Wechsler Adult Intelligence Scale, Third Edition (WAIS-III) [39].

Exclusion criteria included a history of neurological disorders, contraindications to MRI procedures (e.g., claustrophobia, metal implants), pregnancy, and recent caffeine consumption. Participants were instructed to abstain from caffeinated products for at least 12 hours prior to data collection to minimize potential effects on cognitive and neurological function [40]. Normal hearing was confirmed for all participants using the hearWHO application, with acceptable classifications defined as Good or OK based on established thresholds [41],[42].

The study protocol was approved by the human ethics committee USM (USM/JEPeM/21060436). All participants provided written informed consent after receiving comprehensive information about the study procedures, including potential benefits and risks. Participants were assured of their right to withdraw from the study at any point without penalty.

### 2.2. Cognitive flexibility performance assessment

Prior to the auditory stimulation and MRI scanning, participants' cognitive flexibility was assessed using the Working Memory Index subtests from the WAIS-III, which included the Arithmetic, Digit Span, and Letter-Number Sequencing subtests. The Arithmetic subtest required participants to solve 20 arithmetic problems mentally within a time limit, without the use of aids. The Digit Span subtest measured working memory capacity by having participants repeat sequences of numbers in both forward and backward orders. The Letter-Number Sequencing subtest assessed cognitive flexibility by requiring participants to rearrange mixed sequences of letters and numbers, organizing the numbers in ascending order and the letters alphabetically. Participants were required to achieve a working memory index score of 80 or higher to qualify for the experimental stage.

### 2.3. Auditory stimuli

**Figure 1.**
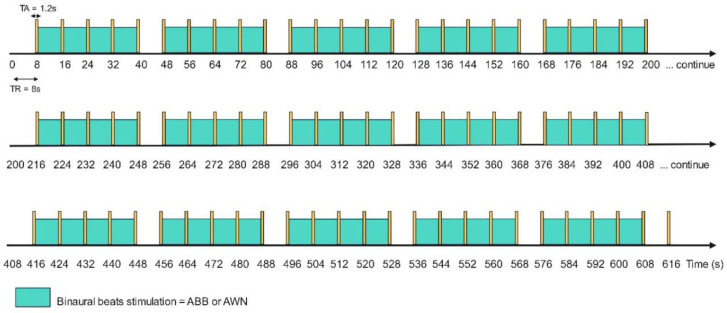
Visual representation of the sparse fMRI paradigm for ABB and AWN.

Two auditory conditions were examined: (1) ABB, characterized by a frequency of 9.55 Hz, produced with carrier tones of 220.45 and 230 Hz, and (2) AWN, identical binaural beats combined with white noise. The auditory stimuli were generated using Audacity 2.3.3 and measured at 75 dB SPL using the Sound Meter app (ver. 1.7.20) by Smart Tools Inc [43]. The stimuli were delivered through high-quality noise-canceling headphones to ensure optimal auditory presentation. The fMRI paradigm design for the sparse sampling method was adapted from Aloysius et al. [20]. Each auditory condition was presented for 8 min per run, with participants completing two runs per session, resulting in a total session duration of 16 min. Figure 1 provides a visual representation of the sparse paradigm for this study.

### 2.4. MRI setup and imaging parameters

Functional MRI data were acquired using a 3.0 T Philips Achieva scanner with a 32-channel head coil to enhance signal-to-noise ratio and spatial resolution. A sparse sampling method was employed to minimize scanner noise interference and preserve auditory processing integrity. Functional images were acquired using an echo-planar imaging (EPI) sequence with the following parameters: sequence type = interleaved, repetition time (TR) = 8 s, echo time (TE) = 30 ms, acquisition time (TA) = 1.2 s, flip angle = 78°, and voxel size = 1.7 × 1.7 × 3 mm. A total of 77 volumes were obtained for each run, utilizing an acquisition matrix of 112 × 112 and a field of view of 192 × 192 mm. High-resolution structural T1-weighted images were acquired using a 3D fast field echo sequence with TR = 7.5 ms, TE = 3.5 ms, flip angle = 8°, and voxel size = 1.0 × 1.0 × 1.2 mm.

### 2.5. Data preprocessing

**Figure 2.**
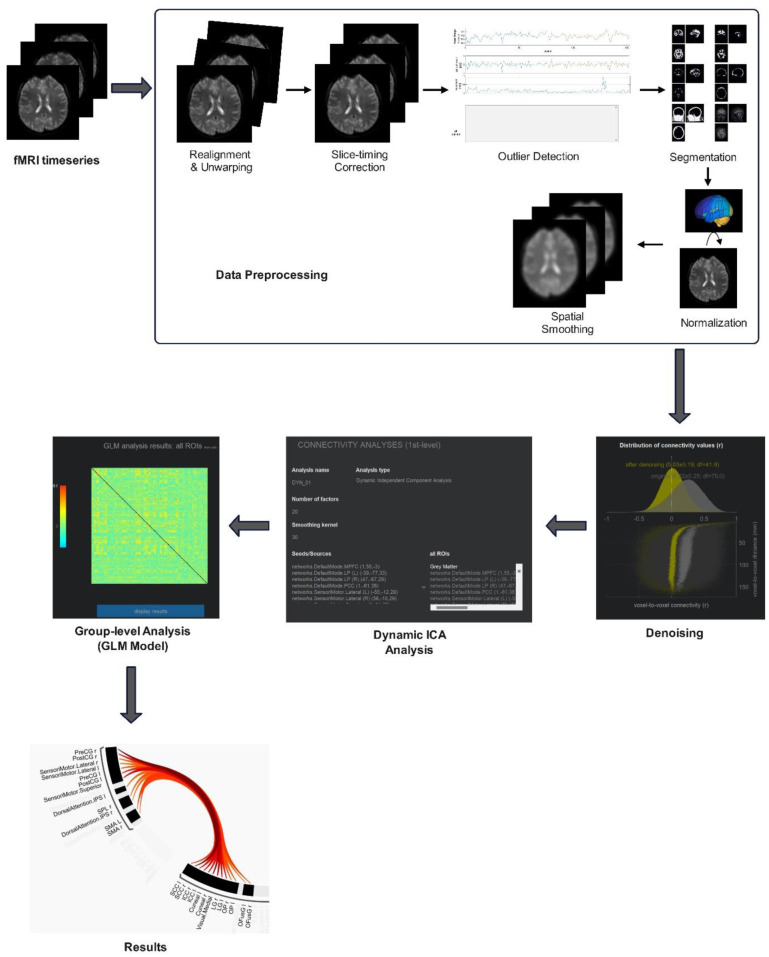
Data analysis pipeline for fMRI preprocessing and dyn-ICA.

Functional and anatomical data were preprocessed using the CONN toolbox [44] and Statistical Parametric Mapping 12 (SPM12). The overall data preprocessing and connectivity analysis workflow is illustrated schematically in Figure 2. Preprocessing steps included realignment and correction for susceptibility distortion using the SPM realign and unwarp procedure [45], slice-timing correction, outlier detection, segmentation, normalization to Montreal Neurological Institute (MNI) space, and smoothing with a Gaussian kernel at full width at half maximum of 6 mm [46]. Temporal misalignment across slices [47],[48], acquired in interleaved Philips order, was corrected using Sinc interpolation. Potential outlier scans were identified based on framewise displacement exceeding 0.9 mm or global signal changes surpassing 5 standard deviations [49],[50]. The functional data were normalized to MNI space, segmented into grey matter, white matter, and cerebrospinal fluid, and resampled to 2 mm isotropic voxels [49],[51].

### 2.6. Denoising

Denoising procedures were performed to minimize confounding influences [44]. These included regressing out noise components associated with white matter and cerebrospinal fluid (5 CompCor components each), motion parameters (12 factors) [52], outlier scans, session effects, and linear trends. A high-pass filter [53] was applied above 0.008 Hz to capture significant changes in the blood-oxygen-level-dependent (BOLD) signal while preserving relevant task signals and ensuring robust statistical validity.

### 2.7. Dynamic independent component analysis (dyn-ICA)

Functional connectivity was examined using dyn-ICA within the CONN toolbox [54]. A total of 20 temporal modulation factors were evaluated to characterize dynamic changes in functional connectivity during auditory stimulation. The analysis modeled interactions between 164 Human Connectome Project ICA networks [54],[55] and regions of interest (ROIs) derived from the Harvard-Oxford atlas, employing a temporal modulation kernel of 30 s to capture patterns of dynamic connectivity. Connectivity among ROIs was measured using a generalized psychophysiological interaction model [56],[57], followed by a fast independent component analysis approach to delineate independent group-level circuits. Participant-specific ICA maps were generated to illustrate connection patterns associated with ABB and AWN.

### 2.8. Statistical analysis at the group level

Group-level analyses were conducted within a general linear model framework [44]. Connectivity measures for each connection were treated as dependent variables, while group identifiers served as independent variables. The statistical significance of the effects related to ABB, AWN, and their average effects was established using a false discovery rate (FDR) correction with a threshold of p < 0.001. This correction was applied to connection-level and cluster-level analyses [multivoxel pattern analysis (MVPA) omnibus test]. For direct comparison of ABB > AWN and AWN > ABB, a more lenient threshold of p < 0.05 (uncorrected) was used for connection-level analyses, while an FDR correction was applied at the cluster level (MVPA omnibus test). This approach balanced sensitivity for detecting subtle differences between conditions with the need to control for multiple comparisons.

## 3. Results

### 3.1. Demographic data

Participant demographics and baseline working memory data were collected and compared between the ABB and AWN groups to assess comparability and confirm eligibility based on inclusion criteria. The working memory index score from the WAIS was used to screen participants based on the study's inclusion criteria. Independent sample t-tests were conducted for continuous variables for working memory index score, while Chi-squared tests were used for categorical variables such as gender and degree year. Due to non-normal distribution observed in the Shapiro–Wilk test for age, a nonparametric Mann–Whitney U test was applied.

The analysis revealed no significant differences in age between the ABB (median 22.00, IQR = 21.0–23.0) and AWN (median = 22.00, IQR = 22.0–23.0) groups (U = 82.50, z = −1.040, p = 0.298). Working Memory Index scores were comparable across groups [ABB: 98.21 ± 16.27; AWN: 96.13 ± 12.79; t(27) = 0.384, p = 0.704], confirming that both groups met the screening criteria similarly. Chi-squared tests demonstrated significant differences in gender distribution (χ^2^ = 0.056, p = 0.812) or academic year (χ^2^ = 0.077, p = 0.782) between the groups. Demographic data for both groups are presented in Table 1. These findings confirm that ABB was well-matched regarding key demographic and baseline cognitive characteristics, supporting a valid comparison of their effects on brain connectivity during auditory stimulation.

**Table 1.** Demographic data.

Variable	Alpha binaural beats (n = 14)	Alpha embedded with white noise (n = 15)	Test statistic	p-value
Age (median, IQR)	22.00 (21.0–23.0)	22.00 (22.0–23.0)	U = 82.50, z = −1.040	0.298
Gender			χ^2^(1) = 0.056	0.812
- Male, n (%)	5 (35.7%)	6 (60.0%)		
- Female, n (%)	9 (64.3%)	9 (40.0%)		
Degree year			χ^2^(1) = 0.077	0.782
- 3rd year, n (%)	10 (71.4%)	10 (66.7%)		
- 4th year, n (%)	4 (28.6%)	5 (33.3%)		
Working Memory Index score (mean ± SD)	98.21 ± 16.27	96.13 ± 12.79	t(27) = 0.384	0.704

### 3.2. Dyn-ICA spatial component analysis

The dyn-ICA identified distinct patterns of brain connectivity modulation associated with ABB and AWN auditory conditions. This analysis revealed multiple functional circuits exhibiting significant changes in connectivity related to auditory stimuli.

### 3.3. Effect of ABB on specific cognitive networks

**Figure 3.**
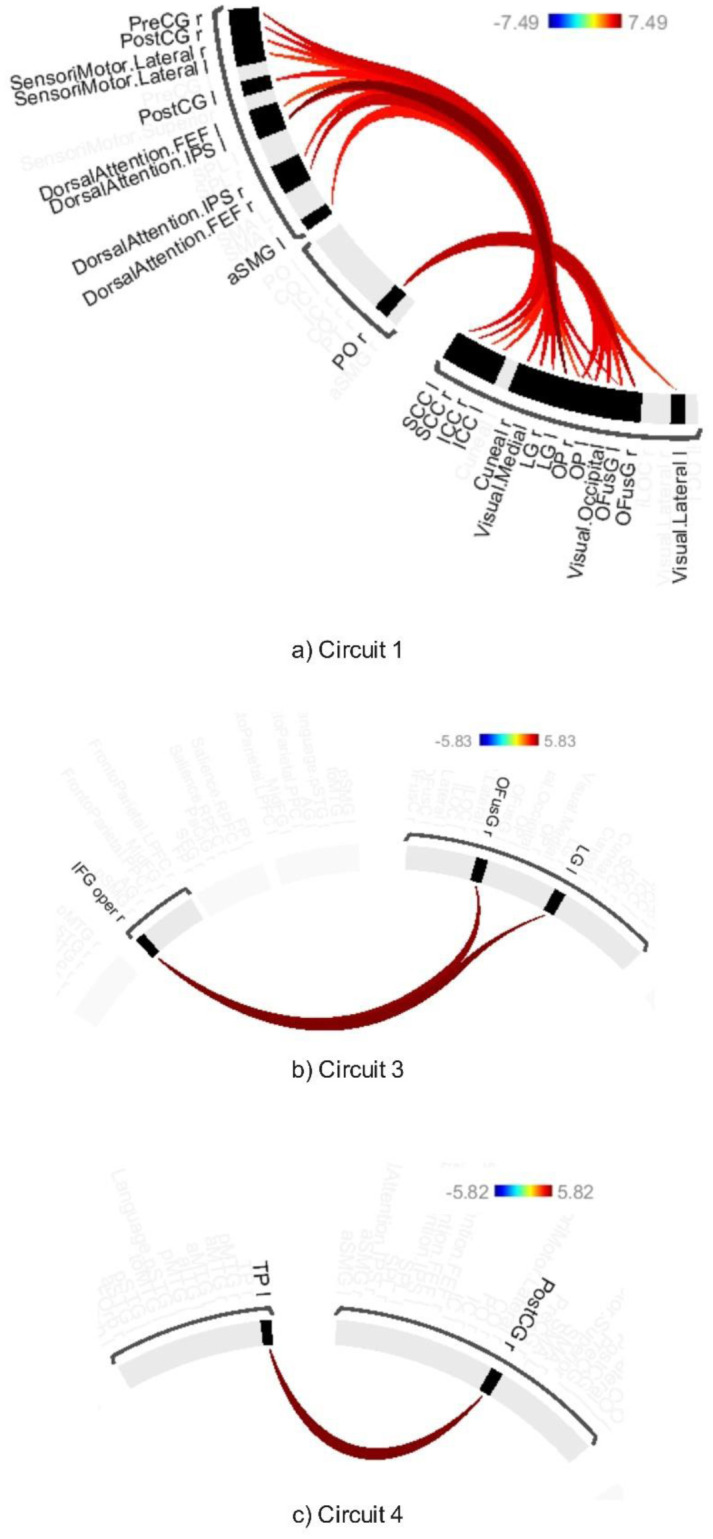
Connectivity pattern for a) Circuit 1, b) Circuit 3, and c) Circuit 4, illustrating the effect of ABB stimulation. Preliminary results were thresholded using a combination of a p-FDR < 0.001 connection-level threshold and a family-wise corrected p-FDR < 0.001 cluster-level threshold (MVPA omnibus test).

**Figure 4.**
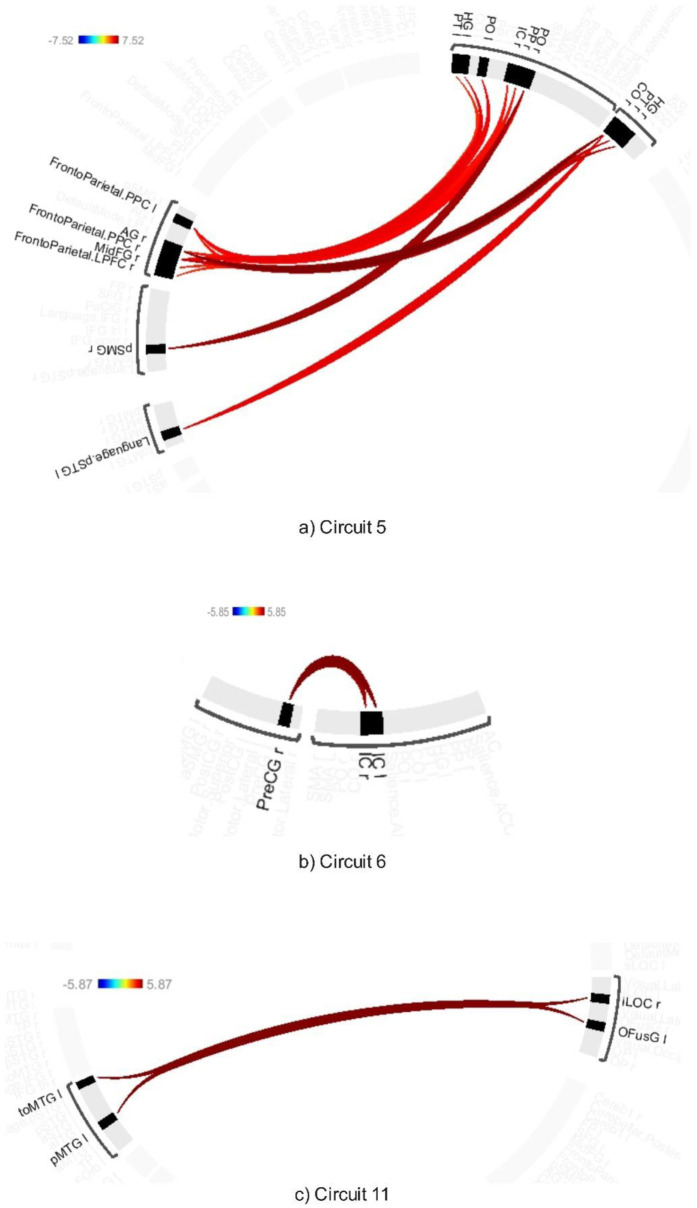
Connectivity pattern for a) Circuit 5, b) Circuit 6, and c) Circuit 11, illustrating the ABB stimulation. Preliminary results were thresholded using a combination of a p-FDR < 0.001 connection-level threshold and a family-wise corrected p-FDR < 0.001 cluster-level threshold (MVPA omnibus test).

ABB significantly modulated connectivity across six circuits (Circuits 1, 3, 4, 5, 6, and 11), as determined by connection-level p-FDR of 0.001 and a cluster-level threshold of p-FDR of 0.001 (MVPA omnibus test). These modulations primarily involved changes in connectivity between visual, attentional, sensorimotor, and cognitive networks (see Figures 3,4 and Appendix 1).

Circuit 1 showed increased connectivity between the lingual gyrus and various regions of the dorsal attention network, including the intraparietal sulcus and the frontal eye fields. Heightened connectivity was observed between visual areas and the sensorimotor cortices, specifically the precentral and postcentral gyri.

Circuit 3 demonstrated increased connectivity between the lingual and inferior frontal gyrus. Circuit 4 revealed enhanced connectivity between the temporal pole and the postcentral gyrus. Circuit 5 exhibited increased connectivity across several regions, including the right angular gyrus, right central opercular cortex, right Heschl's gyrus, right planum temporale, and portions of the posterior parietal and lateral prefrontal cortex within the frontoparietal networks.

Circuit 6 displayed enhanced connectivity between the precentral gyrus and the supramarginal gyrus. Additionally, Circuit 11 showed increased connectivity between the lateral occipital cortex and the middle temporal gyrus.

### 3.4. AWN-related modulation of visual and attentional networks

**Figure 5.**
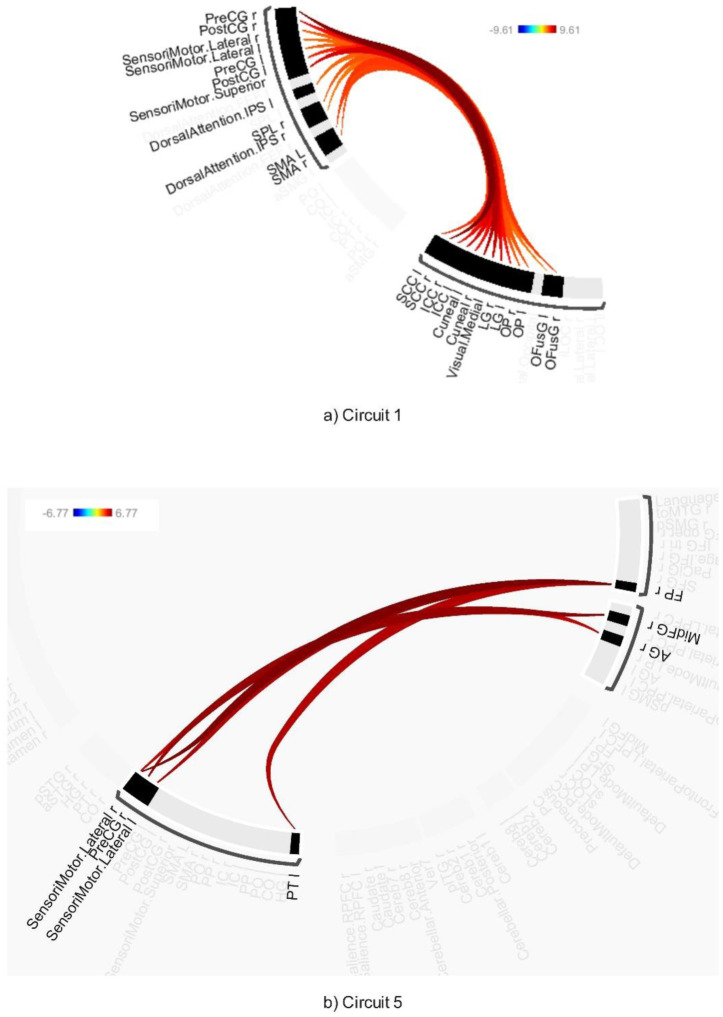
Connectivity pattern for a) Circuit 1 and b) Circuit 5, illustrating the effect of AWN stimulation. Preliminary results were thresholded using a combination of a p-FDR < 0.001 connection-level threshold and a family-wise corrected p-FDR < 0.001 cluster-level threshold (MVPA omnibus test).

**Figure 6.**
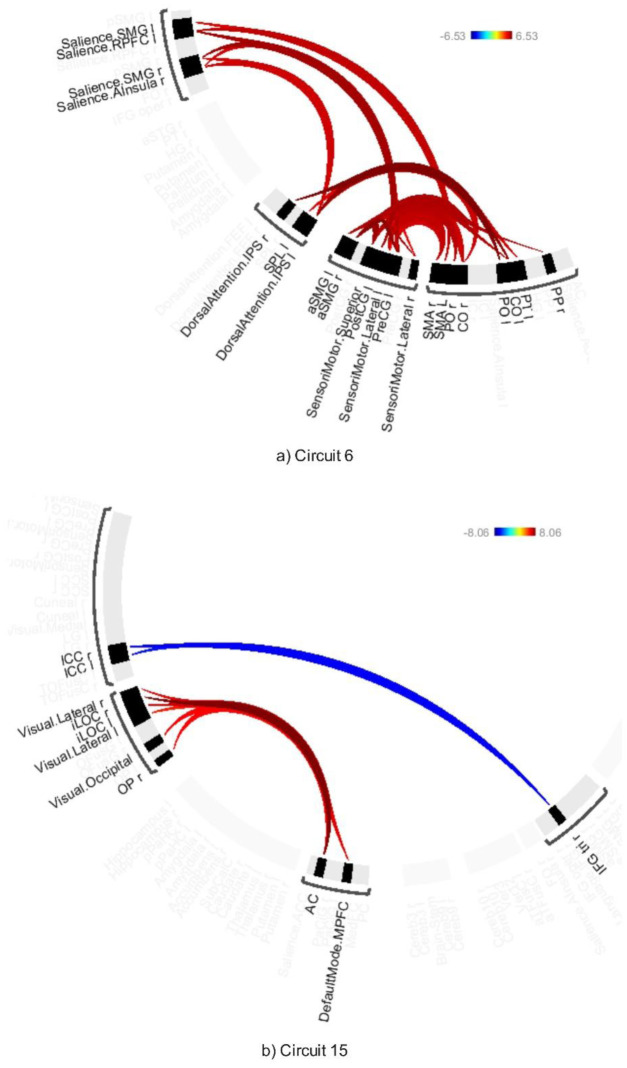
Connectivity pattern for a) Circuit 6 and b) Circuit 15, illustrating the effect of AWN stimulation. Preliminary results were thresholded using a combination of a p-FDR < 0.001 connection-level threshold and a family-wise corrected p-FDR < 0.001 cluster-level threshold (MVPA omnibus test).

AWN significantly modulated connectivity within four identified circuits [Circuits 1, 5, 6, and 15 (see Figures 5,6 and Appendix 2)] at a connection-level threshold of p-FDR < 0.001 and a cluster-level threshold of p-FDR < 0.001 (MVPA omnibus test).

Circuit 1 demonstrated increased connectivity between the precentral gyrus and various visual areas, including the supracalcarine cortex, cuneus, and lingual gyrus. Circuit 5 showed heightened connectivity between the precentral gyrus and the middle frontal gyrus, as well as between the angular gyrus and lateral sensorimotor regions, and between the frontal pole and sensorimotor areas. Circuit 6 revealed increased connectivity between the precentral gyrus and the supramarginal gyrus.

In Circuit 15, it was found that AWN was associated with increased connectivity between the lateral occipital cortex and the anterior cingulate gyrus, as well as greater connectivity between the default mode network and visual areas. Additionally, negative connectivity was observed between the inferior frontal gyrus and the intracalcarine cortex.

### 3.5. Differential effect of AWN and ABB on emotional regulation

**Figure 7.**
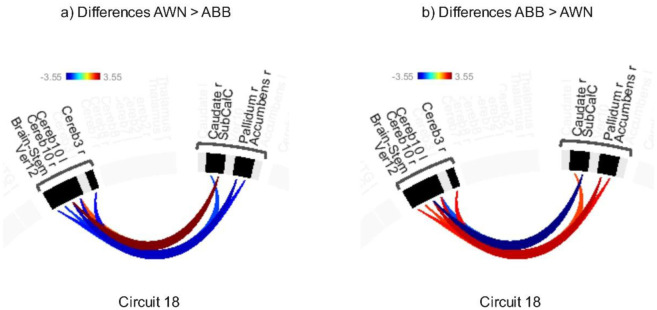
Difference of (a) AWN > ABB and (b) ABB > AWN thresholded using a combination of a p-FDR < 0.05 connection-level threshold and a corrected p-FDR < 0.05 cluster-level threshold (MVPA omnibus test).

The AWN > ABB contrast showed modulated connectivity in Circuit 18 (Figure 7a; Appendix 3a) thresholded at an uncorrected connection-level threshold of p < 0.05 and a corrected cluster-level threshold of p-FDR < 0.05 (MVPA omnibus test), with increased connectivity between the cerebellum and subcallosal cortex, and decreased connectivity between the accumbens and cerebellum.

Conversely, the ABB > AWN contrast showed modulated connectivity in Circuit 18 (Figure 7b; Appendix 3b), revealing increased connectivity between the cerebellum and pallidum, and between the vermis and the accumbens and caudate, along with decreased connectivity between the cerebellum and subcallosal cortex.

### 3.6. Average effects of ABB and AWN on brain connectivity

**Figure 8.**
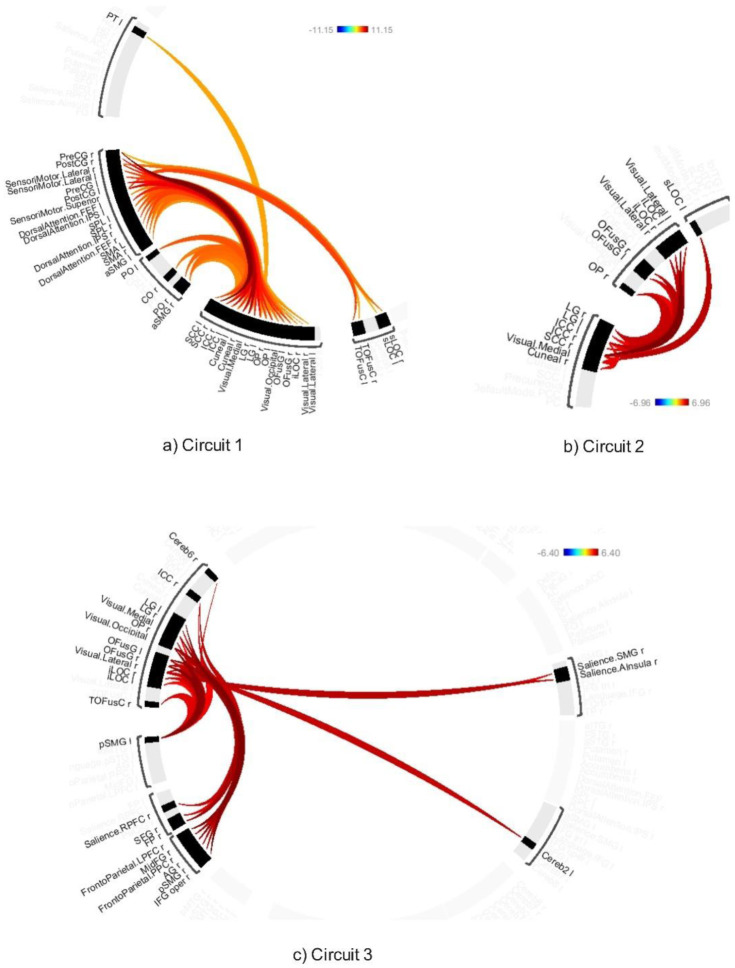
Connectivity pattern for a) Circuit 1, b) Circuit 2, and c) Circuit 3, illustrating the average effects of AWN and ABB stimulation. Preliminary results were thresholded using a combination of a p-FDR < 0.001 connection-level threshold and a family-wise corrected p-FDR < 0.001 cluster-level threshold (MVPA omnibus test).

**Figure 9.**
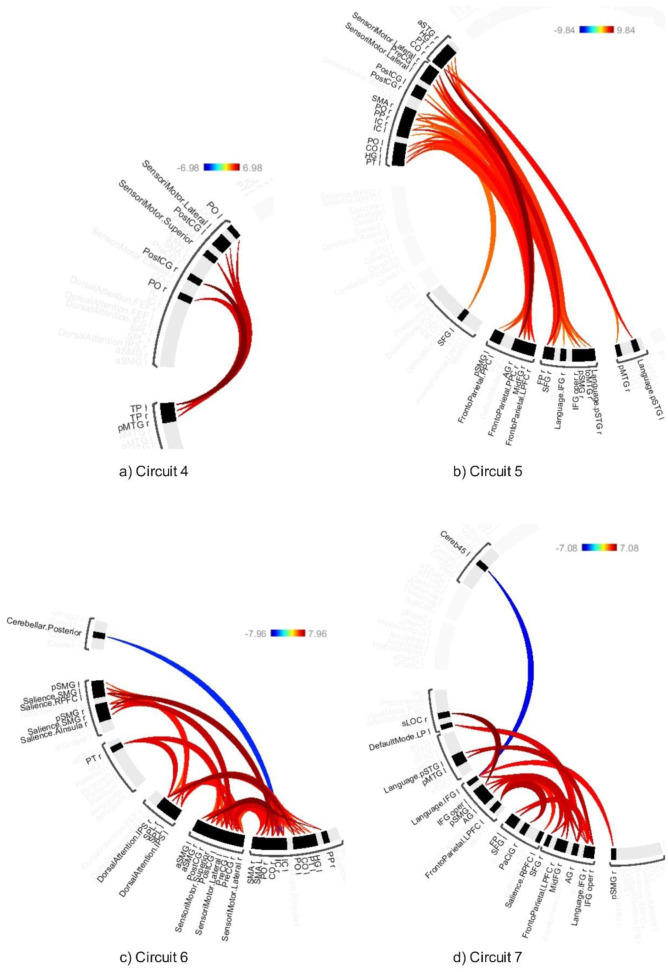
Connectivity pattern for a) Circuit 4, b) Circuit 5, c) Circuit 6, and d) Circuit 7, illustrating the average effects of AWN and ABB stimulation. Preliminary results were thresholded using a combination of a p-FDR < 0.001 connection-level threshold and a family-wise corrected p-FDR < 0.001 cluster-level threshold (MVPA omnibus test).

**Figure 10.**
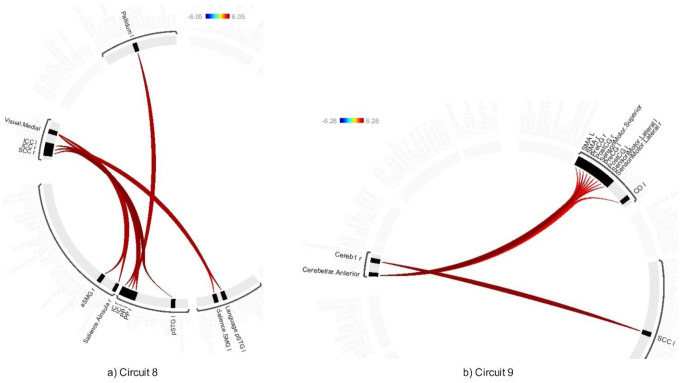
Connectivity pattern for a) Circuit 10 and b) Circuit 9, illustrating the average effects of AWN and ABB stimulation. Preliminary results were thresholded using a combination of a p-FDR < 0.001 connection-level threshold and a family-wise corrected p-FDR < 0.001 cluster-level threshold (MVPA omnibus test).

**Figure 11.**
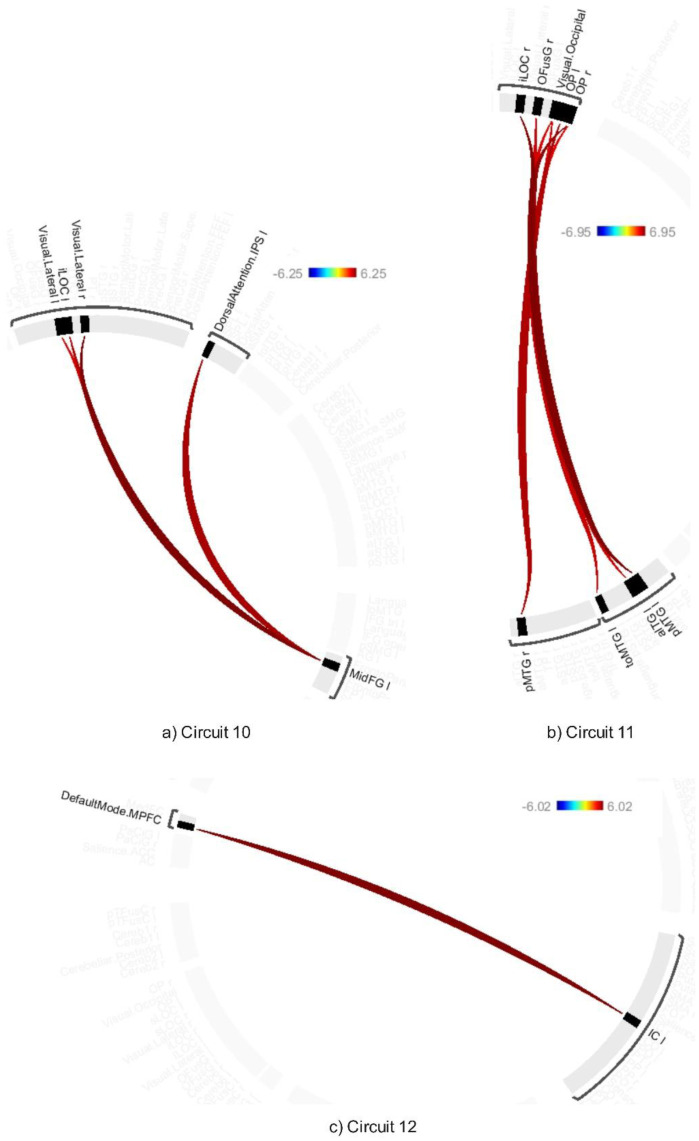
Connectivity pattern for a) Circuit 10, b) Circuit 11, and c) Circuit 12, illustrating the average effects of AWN and ABB stimulation. Preliminary results were thresholded using a combination of a p-FDR < 0.001 connection-level threshold and a family-wise corrected p-FDR < 0.001 cluster-level threshold (MVPA omnibus test).

**Figure 12.**
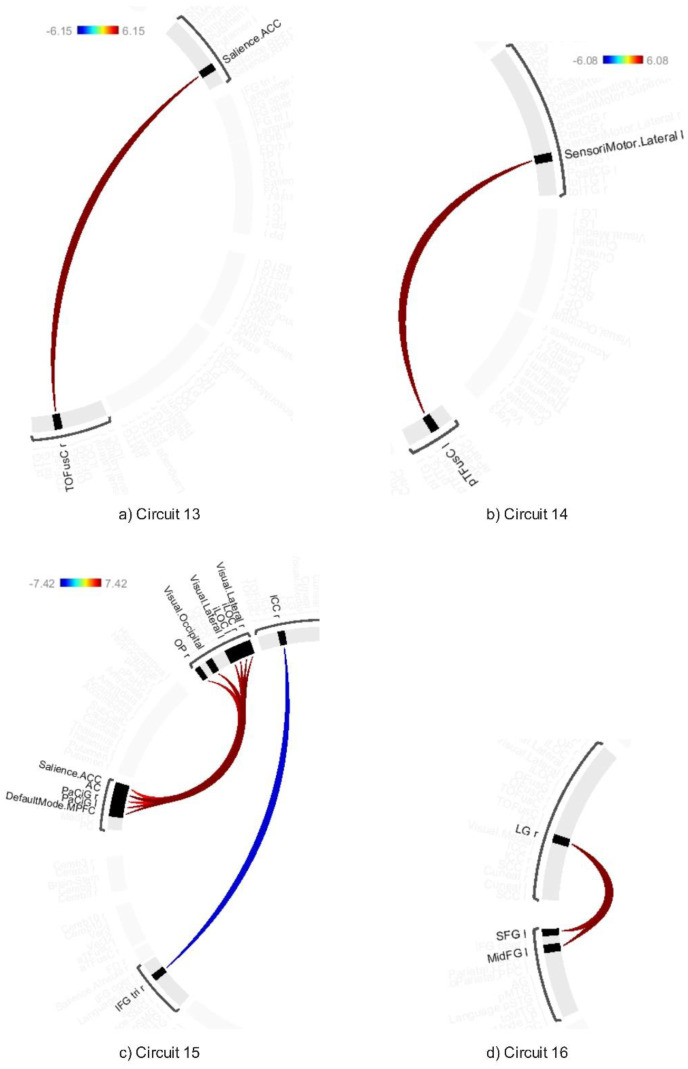
Connectivity pattern for a) Circuit 13, b) Circuit 14, c) Circuit 15, and d) Circuit 16, illustrating the average effects of AWN and ABB stimulation. Preliminary results were thresholded using a combination of a p-FDR < 0.001 connection-level threshold and a family-wise corrected p-FDR < 0.001 cluster-level threshold (MVPA omnibus test).

**Figure 13.**
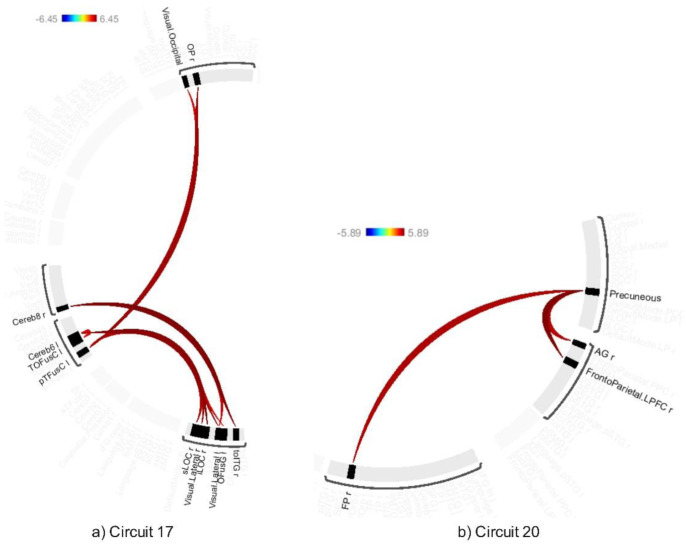
Connectivity pattern for a) Circuit 13, b) Circuit 14, c) Circuit 15, and d) Circuit 16, illustrating the average effects of AWN and ABB stimulation. Preliminary results were thresholded using a combination of a p-FDR < 0.001 connection-level threshold and a family-wise corrected p-FDR < 0.001 cluster-level threshold (MVPA omnibus test).

The average effects of ABB and AWN revealed significant connectivity changes across 18 circuits (1–17 and 20) at a connection-level threshold of p-FDR < 0.001 and a cluster-level threshold of p-FDR < 0.001 (MVPA omnibus test) (see Figures 8–13 and Appendix 4). These exhibited distinct patterns across circuits, indicating the average effects of ABB and AWN on brain connectivity.

Circuit 1 showed increased positive connectivity between the precentral gyrus and regions including the supracalcarine cortex, cuneal cortex, and visual medial network. Circuit 2 demonstrated enhanced connectivity between the supracalcarine cortex, lateral occipital cortex, and intracalcarine cortex. Across the 18 circuits, the combined influence of ABB and AWN was associated with increased connectivity within and between several networks. These included visual and motor systems (Circuits 1, 2, and 17), attentional networks (Circuits 1, 3, 6, 10, and 16), frontoparietal networks (Circuits 3, 5, 7, and 10), different sensory regions (Circuits 5, 13, and 14), and the default mode network and its connections (Circuits 8, 12, 15, and 20).

## 4. Discussion

This exploratory study utilized fMRI and dyn-ICA to investigate how ABB and AWN auditory stimulation modulate functional brain connectivity in young adults during passive listening. Our work provides initial neurobiological insights into how non-invasive auditory stimuli might influence brain network dynamics underlying human behavior, moving from theory toward potential practice. Given the often inconsistent behavioral and brain imaging findings in the existing literature on binaural beats [25],[29],[30], understanding the immediate impact of these stimuli on the underlying brain network is essential. Our findings revealed distinct and overlapping patterns of connectivity modulation across various functional brain circuits. These preliminary neural findings align with the general notion that binaural beats and white noise can induce changes in brain activity [21],[24] but must be interpreted cautiously within the context of the mixed behavioral landscape. The observed network modulations suggest potential underlying neural mechanisms by which these auditory stimuli could influence cognitive states and functions. However, whether and how these neural changes translate to reliable effects, particularly positive ones, remains a key question given the variability in prior outcome studies.

### 4.1. ABB: neural modulations potentially associated with enhanced cognitive flexibility

Young adults, whether in university or starting their careers, often encounter situations that require adaptability and efficient information processing, skills essential for learning, skill development, and achievement. Our preliminary findings characterize the neural modulations induced by ABB that may underlie cognitive capacities, aligning with previous studies suggesting that ABB could influence cognitive flexibility and facilitate switching between different cognitive sets [21],[24].

The enhanced connectivity observed in Circuit 1 between the lingual gyrus (a visual processing region) and regions of the dorsal attention network, including the intraparietal sulcus and the frontal eye fields, suggests that acute ABB stimulation impacts networks involved in visual processing and attention allocation [27],[58]–[64]. Although not directly tested with a concurrent cognitive task, this modulation pattern is consistent with neural states potentially associated with improved visual attention or preparedness for processing external visual information [27],[58]–[64]. This capability is foundational for learning and achievement in visually rich environments, such as reading and processing diagrams [27],[65],[66]. Additionally, the enhanced connectivity observed within Circuit 1 between visual areas and sensorimotor cortices indicates that ABB might influence the integration of visual inputs with motor planning or execution streams [27]. This finding aligns with previous research showing that ABB can enhance alpha-band coherence during visual-motor tasks and visual feedback [63],[64]. Such preliminary neural integration patterns could potentially support behaviors requiring visual-motor coordination, which are relevant for skill development and motion-related tasks (for example, artists, musicians, surgeons, mechanics, and pilots), although this behavioral link was not assessed in the current study.

Circuit 3 showed enhanced connectivity between the lingual and inferior frontal gyrus, suggesting that ABB may promote interactions between visual processing areas and regions associated with executive control. This connectivity pattern could potentially support visual processing during goal-directed activities such as problem-solving and decision-making. This is particularly relevant for young adults in academic and professional settings, where achievement depends on navigating complex challenges. Moreover, the enhanced connectivity between the temporal pole and postcentral gyrus (Circuit 4) suggests that ABB might integrate sensory input with social-emotional information. Thus, integration could be important for relationships and well-being in social contexts relevant to young adults [67]–[69], such as group projects, presentations, and networking events.

Our preliminary findings also suggest that ABB acutely influences networks that integrate information across different sensory modalities. The enhanced connectivity observed in the angular gyrus, the insular cortex, and the lateral sensorimotor regions within Circuit 5 indicates that ABB may promote functional communication among areas related to auditory, visual, and somatosensory [70],[71]. This preliminary cross-modal integration is crucial for effective learning, comprehension, and adaptation to new environments and tasks.

Taken together, the acute network modulation changes induced by ABB, which involve increased connectivity within and between attentional, visual, sensorimotor, and potentially integrative circuits, indicate a neural state that could potentially support cognitive flexibility and complex processing. This aligns with brain states relevant to various aspects of learning, skill development, achievement, and interpersonal relationships.

### 4.2. AWN: enhancing focus and reducing distractions in demanding environments

Young adults often face environments filled with distractions, such as open-plan offices, busy cafes, or shared living spaces. This distraction can pose significant challenges for learning and achievement. Our preliminary findings suggest that the neural modulations induced by AWN may be associated with enhanced focused processing and potentially reduced susceptibility to distractions, thereby supporting achievement in challenging environments.

The increased connectivity observed in Circuit 1 between sensorimotor and visual regions highlights the influence of AWN on these networks, potentially enhancing visual-motor integration. This preliminary neural pattern is relevant for tasks that require focused attention and precise motor control [71]–[73], such as writing and typing. It aligns with the understanding of the role of visual-motor integration in attention and cognitive regulation [72],[74],[75], which is essential for skill development and achievement. These benefits are particularly important for young adults in various professions that demand sustained attention and fine motor skills, including programmers, writers, and designers.

Furthermore, the preliminary modulation of connectivity in networks related to executive control and sensory integration (Circuits 5 and 6) by AWN suggests that this stimulation may impact how information is processed in complex multisensory environments. This could potentially support enhanced attention [33],[76],[77], a core component of learning and achievement. These findings are consistent with research suggesting that white noise can influence cognitive performance, possibly by filtering distractions [32],[33],[35],[78]. This could be especially beneficial for young adults working in open-plan offices or those who need to focus on complex tasks in a busy environment.

In Circuit 15, the enhanced connectivity between the lateral occipital cortex and the anterior cingulate gyrus, as well as the connections between the default mode network and visual areas, suggests that AWN stimulation may influence networks potentially involved in sustained attention [79]–[81], visual processing [81]–[83], and memory retrieval [84],[85]. This relationship is important for balancing mind-wandering and task engagement, which are crucial for sustained achievement. These functions are crucial for academic success and professional performance, especially for tasks that require prolonged focus and visual attention [86],[87] such as data analysis, document review, or conducting research.

The changes induced by AWN stimulation may affect networks associated with visual-motor integration, executive control, attentional networks, interaction within the default mode network, and limbic or reward networks. This suggests a preliminary neural state that could support focused processing. Consequently, it may influence achievement, specific aspects of skill development, and overall well-being by altering attention and emotional regulation states.

### 4.3. Shared mechanisms of ABB and AWN: enhancing cognitive control and attention

While ABB and AWN exhibit distinct modulation patterns, the average effect analysis suggests that auditory stimuli also engage common pathways associated with fundamental cognitive functions. Specifically, both ABB and AWN were preliminarily found to enhance connectivity within the frontoparietal network, a core system associated with cognitive control, working memory, and flexible decision making [88]–[90]. Strengthening connectivity in this network may indicate an immediate influence of auditory stimulation on brain states that are crucial for learning, skill development, and the regulation of goal-directed behavior.

In addition to frontoparietal modulation, both auditory stimuli demonstrated shared influence on networks involved in visual attention and processing efficiency [91],[92]. Enhanced connectivity within visual and attention-related networks [91],[92] may facilitate more efficient sensory integration and attentional allocation, processes that are essential for effective interaction with a dynamic environment and for supporting complex behavioral responses. These common preliminary neural substrates could underpin the general effects of auditory stimulation on cognitive processing readiness relevant to a wide range of human behaviors.

Together, these findings suggest that, despite their distinct profiles, ABB and AWN may converge on common neural substrates that acutely prepare the brain for enhanced cognitive readiness. The modulation of frontoparietal and visual-attention networks by both stimuli may be key to understanding the general effects of auditory stimulation on attentional control, adaptive processing, and environmental engagement, supporting cognitive flexibility and performance across various contexts.

### 4.4. Differential effects of ABB and AWN: implications for tailoring potential brain–behavior interventions

The distinct neural modulation patterns observed for AWN and ABB highlight their complementary roles in modulating brain activity and cognitive states. The differences highlighted, particularly Circuit 18, involve areas associated with motor control, reward processing, and emotional regulation, processes that are crucial for motivation, well-being, and guiding goal-directed behavior.

The differential connectivity patterns in Circuit 18 suggest that AWN and ABB uniquely influence networks related to acute arousal, motivation, or emotional state. This, in turn, could affect cognitive processes such as sustained attention, which is important for achieving goals, and cognitive flexibility, relevant for skill development. While the precise functional consequence of these differential patterns requires further investigation, these preliminary findings indicate that ABB and AWN do not merely induce generic changes in brain process; instead, they may induce specific network reconfigurations in response to stimuli [19],[25],[27],[93],[94].

This supports the idea that these auditory stimuli could potentially be strategically employed to influence brain states that are conducive to achieving specific behavioral goals. For example, AWN could promote a state that fosters focused achievement, while ABB might enhance cognitive flexibility necessary for complex skill development or social interactions. These preliminary neural signatures provide initial neurobiological support for the potential of tailoring auditory stimulation according to the neural states underlying specific behaviors. However, it is important to interpret these findings with caution, as they require validation in future studies involving larger datasets and a diverse range of populations.

### 4.5. Connecting preliminary neural findings to broader theoretical frameworks

Our preliminary findings on how specific auditory stimuli modulate large-scale brain network connectivity offer initial neural insights into the inconsistent behavioral literature and their link with behavior when interpreted within theoretical frameworks. The effect of ABB suggests an increase in positive connectivity within and between attentional, visual, and frontoparietal networks. This indicates a preliminary pattern of enhanced integration, which aligns with the global workspace theory [95],[96]. This integration is crucial for conscious awareness and cognitive flexibility [95],[96], both of which are relevant for learning and achievement. In contrast, the modulation pattern associated with AWN is involved in attention and visual networks, as well as interactions with the default mode network and differential effects on subcortical and cerebellar networks. This aligns with the predictive coding framework [97]–[99], suggesting that auditory input may influence the balance between processing sensory information and internal predictive models. This influence can affect focused attention and the gating of information flow, processes that are central to skill development and achievement [97]–[99]. By situating our findings within these frameworks, we emphasize how ABB and AWN may interact differently with fundamental brain principles over a short timescale. These preliminary neural modulations could contribute to the variability observed in behavioral outcomes and provide initial neurobiological hypotheses regarding the brain basis of specific behaviors that can be targeted through auditory stimulation [97]–[99].

### 4.6. Practical implications and personalized interventions for young adults

The distinctive and complementary neural modulation patterns induced by AWN and ABB highlight their significant potential as tools to influence cognitive function in young adults. These preliminary findings provide an initial neurobiological basis for exploring potential personalized interventions aimed at supporting specific cognitive functions.

ABB appears to induce neural patterns associated with enhanced integration across networks relevant for cognitive flexibility and complex processing. This includes working memory, visual-motor integration, and multisensory processing, processes that are essential for academic success and professional performance. As such, ABB may facilitate improvement in these areas. Conversely, AWN induces neural patterns that may enhance focus and alter the balance between internal and external attention. This involves visual, sensorimotor, executive, and default mode networks. These processes support factors related to motivation and well-being through the modulation of the limbic network. Therefore, AWN has the potential to enhance sustained attention, reduce distractions, improve emotional regulation, and refine visual-motor integration and sensory processing, which can benefit young adults in demanding learning and work environments.

Future research could aim to develop personalized interventions that integrate AWN and ABB, considering individual differences that influence responsiveness to auditory stimulation. This may involve creating algorithms or assessment tools designed to identify optimal stimulation parameters tailored to individuals' needs and characteristics. Combining auditory stimulation via ABB or AWN with real-time neurofeedback represents a promising possibility for personalized neuromodulation. This approach could support specific brain–behavior connections, allowing individuals to self-regulate their cognitive states and potentially enhance the effectiveness of auditory stimuli.

### 4.7. Limitations and future directions

This study offers valuable preliminary insights into the effects of ABB and AWN on brain connectivity. However, several limitations must be acknowledged. First, the sample size is relatively small (n = 29; ABB = 14, AWN = 15), and the slight imbalance between the groups limits the statistical power to detect subtle effects, thereby constraining the generalizability of the findings. Although our sample size aligns with methodological recommendations for pilot or preliminary studies aimed at informing future research [38], this modest cohort necessitates a cautious interpretation of the results. Notably, despite the small sample, the brain network connections demonstrated large effect sizes (see Appendices 1–4), suggesting robust underlying patterns that warrant further investigation. These findings highlight the necessity for larger, adequately powered studies to validate the preliminary patterns observed in this study.

Second, the exclusion of young adult participants restricts the generalizability of the findings across broader age groups. Given known age-related variations in brain function and cognitive processes, future studies should explore the effects of ABB and AWN across different stages of life to enhance the applicability of auditory stimulation interventions. Moreover, this study did not directly evaluate the effects of ABB and AWN on specific cognitive tasks.

Third, although this study characterized neural modulation during binaural beats listening, it did not incorporate concurrent behavioral assessments to establish a direct link between changes in brain connectivity and cognitive or emotional outcomes. To more convincingly connect the observed neural modulations to cognitive outcomes, future research must incorporate targeted behavioral assessments. For example, tasks that evaluate specific aspects of attention (e.g., sustained attention and selective attention), cognitive flexibility (e.g., task-switching paradigms), and working memory (e.g., n-back tasks) would provide crucial validation of the neural findings. Additionally, although our discussion offers plausible interpretations regarding cognitive flexibility, emotional regulation, and multisensory integration based on known neuroanatomical functions, these remain speculative in the absence of direct behavioral or physiological measures targeting these specific constructs. Future research should utilize experimental designs specifically aimed at testing these interpretations.

Fourth, the absence of a passive listening control condition (e.g., silence or sham sound) limits the ability to isolate the specific effects of ABB and AWN from general auditory influences. Future studies should incorporate appropriate control conditions to more precisely attribute observed neural changes to the auditory stimuli.

Additionally, this research only assessed the immediate effects of ABB and AWN. Longitudinal studies are needed to evaluate the long-term impacts of repeated ABB and AWN stimuli on brain connectivity and cognitive performance over time. Combining ABB and AWN with other interventions, such as neurofeedback or cognitive training, is promising for enhancing cognitive outcomes. Furthermore, examining individual differences, including baseline brain connectivity, cognitive profiles, personality traits, and genetic predispositions, may inform the development of personalized auditory stimulation strategies. Despite these limitations, the present study provides foundational data to inform future, more definitive investigations into the potential of auditory stimulation for cognitive and emotional modulation.

## 5. Conclusion

This study uses dyn-ICA analysis to examine in depth how auditory stimuli characterized as ABB and AWN affect functional brain connectivity in young adults. Our preliminary findings reveal distinct and complementary neural modulation patterns across various functional brain networks, offering initial insights into the neurobiological basis of how these auditory stimuli may influence brain states relevant to human behavior.

Specifically, AWN significantly enhanced connectivity within sensorimotor, visual, and executive control networks. These results suggest that AWN has the potential to modulate focused cognitive processing, enhance sensory integration, enhance attentional control, and decrease susceptibility to distractions. This indicates that AWN may be particularly advantageous for young adults in high-demand learning and work environments, where sustained attention, visual-motor coordination, and emotional regulation are crucial.

In contrast, ABB facilitated greater integration and communication across a broader range of brain networks, including the frontoparietal network, visual-motor networks, and circuits involved in multisensory integration. This enhanced integration likely promotes cognitive flexibility, robust attention control, and efficient information processing, thereby contributing to more adaptive and goal-directed behaviors essential for academic success and professional development.

The implications of these findings are substantial for the development of personalized cognitive interventions tailored to distinct cognitive demands. By clarifying the distinct impacts of AWN and ABB on brain connectivity, interventions can be customized to address individual cognitive needs and objectives. Notably, individuals with attention deficits or those in high-pressure environments may significantly benefit from AWN to enhance focus and reduce distractions. Conversely, ABB may be utilized to enhance cognitive flexibility and optimize performance in tasks necessitating creativity, problem-solving, and multitasking. These insights pave the way for utilizing auditory stimulation as a non-invasive method to support cognitive optimization across educational, occupational, and therapeutic contexts.

Nevertheless, it is important to recognize that this study is exploratory and based on a small sample size with slight group imbalances, which limit statistical power and generalizability. Furthermore, given that prior research on the cognitive and neural effects of binaural beats has produced inconsistent and mixed results, the current findings should be interpreted cautiously. They are best viewed as generating hypotheses rather than definitive conclusions.

Future research should include concurrent behavioral assessments, longitudinal designs, appropriate control groups, and investigations into individual differences to validate and extend these preliminary observations. Larger, rigorously conducted studies are essential to clarify the long-term effects of ABB and AWN, define their influence on specific cognitive outcomes, and establish their role in targeted cognitive and behavioral interventions. These efforts are essential to move from preliminary insights toward evidence-based applications of auditory stimulation strategies for cognitive enhancement and emotional well-being.

## Use of AI tools declaration

The authors declare they have not used Artificial Intelligence (AI) tools in the creation of this article.


