# Supplementary material for: Distinct neural mechanisms of alpha binaural beats and white noise for cognitive enhancement in young adults
Source: AIMS Neurosci. 2025 May 20;12(2):147–79. doi: 10.3934/Neuroscience.2025010 (PMC12287642; doi:10.3934/Neuroscience.2025010)
Supplement: Supplementary file 1 [file neurosci-12-02-010-s001.pdf]

AIMS Neuroscience

AIMS Neuroscience, 12(2): 147–179.
DOI: 10.3934/Neuroscience.2025010
Received: 06 February 2025
Revised: 29 April 2025
Accepted: 15 May 2025
Published: 20 May 2025

https://www.aimspress.com/journal/neuroscience

Research article

Distinct neural mechanisms of alpha binaural beats and white noise for cognitive enhancement in young adults

Aini Ismafairus Abd Hamid[1,2,3,*], Nurfaten Hamzah[1,2], Siti Mariam Roslan[1], Nur Alia Amalin Suhardi[4], Muhammad Riddha Abdul Rahman[1,5], Faiz Mustafar[1,2], Hazim Omar[1,2,3], Asma Hayati Ahmad[2,3,6], Elza Azri Othman[5] and Ahmad Nazlim Yusoff[7]

[1] Department of Neurosciences, School of Medical Sciences, Health Campus, Universiti Sains Malaysia, Kubang Kerian, Kelantan, Malaysia
[2] Brain & Behaviour Cluster, School of Medical Sciences, Universiti Sains Malaysia, Kubang Kerian, Kelantan, Malaysia
[3] Hospital Pakar Universiti Sains Malaysia, Health Campus, Universiti Sains Malaysia, Kubang Kerian, Kelantan, Malaysia
[4] Faculty of Cognitive Sciences and Human Development, Universiti Malaysia Sarawak, Kota Samarahan, Sarawak, Malaysia
[5] School of Medical Imaging, Faculty of Health Sciences, Universiti Sultan Zainal Abidin, Kuala Nerus, Terengganu, Malaysia
[6] Department of Physiology, School of Medical Sciences, Health Campus, Universiti Sains Malaysia, Kubang Kerian, Kelantan, Malaysia
[7] Center for Diagnostic, Therapeutic and Investigative Studies, Faculty of Health Science, Universiti Kebangsaan Malaysia, Jalan Raja Muda Abdul Aziz, Wilayah Persekutuan Kuala Lumpur, Malaysia

* **Correspondence:** Email: aini_ismafairus@usm.my; Tel: +6097676300.

Supplementary material

Appendix 1. Effect of alpha binaural beats (ABB) thresholded using a combination of a p-FDR < 0.001 connection-level threshold and a corrected p-FDR < 0.001 cluster-level.

Circuit	Analysis Unit	Statistic	p-unc	p-FDR	Effect Size (Hedges' g)
1	***Cluster 1/78***	***F(2,26) = 42.72***	***0.000000***	***0.000000***	2.804
	Left Lingual Gyrus-Dorsal Attention Networks (Left Intraparietal Sulci)(−39, −43,52)	T(27) = 7.49	0.000000	0.000008	2.384
	Left Lingual Gyrus-Dorsal Attention Networks (Right Frontal Eye Fields) (30, −6,64)	T(27) = 6.37	0.000001	0.000066	2.265
	Medial Visual Networks (2, −79, 12)-Dorsal Attention Networks (Right Frontal Eye Fields)(30, −6, 64)	T(27) = 6.05	0.000002	0.000155	2.261
	Medial Visual Networks (2, −79, 12)-Precentral Gyrus Right	T(27) = 6.04	0.000002	0.000155	2.197
	Medial Visual Networks (2, −79, 12)-Postcentral Gyrus Right	T(27) = 5.87	0.000003	0.000163	2.328
	Right Precentral Gyrus-Right Supracalcarine Cortex	T(27) = 6.22	0.000001	0.000187	2.182
	Right Precentral Gyrus-Left Occipital Pole	T(27) = 5.83	0.000003	0.000187	2.179
	Right Precentral Gyrus-Medial Visual Networks (2, −79, 12)	T(27) = 5.82	0.000003	0.000187	2.137
	Medial Visual Networks (2, −79, 12)-Dorsal Attention Networks (Left Intraparietal Sulci)(−39, −43, 52)	T(27) = 5.71	0.000005	0.000188	2.122
	Right Precentral Gyrus-Right Cuneal Cortex	T(27) = 5.67	0.000005	0.000209	2.089
	Medial Visual Networks (2, −79, 12)-Left Postcentral Gyrus	T(27) = 5.58	0.000006	0.000209	2.021
	Medial Visual Networks (2, −79, 12)-Anterior Division Left Supramarginal Gyrus	T(27) = 5.40	0.000010	0.000251	2.018
	Medial Visual Networks (2, −79, 12)-Right Lateral Sensorimotor Networks (56, −10, 29)	T(27) = 5.39	0.000011	0.000251	2.227
	Right Postcentral Gyrus-Left Occipital Fusiform Gyrus	T(27) = 5.95	0.000002	0.000392	2.804
	Right Precentral Gyrus-Left Intracalcarine Cortex	T(27) = 5.31	0.000013	0.000427	1.988
	Right Lingual Gyrus-Dorsal Attention Networks (Right Frontal Eye Fields) (30, −6, 64)	T(27) = 5.88	0.000003	0.000469	2.201
	Left Supracalcarine Cortex-Dorsal Attention Networks (Left Intraparietal Sulci) (−39, −43, 52)	T(27) = 5.86	0.000003	0.000500	2.194

	Left Lingual Gyrus-Dorsal Attention Networks (Right Intraparietal Sulci) (39, −42, 54)	T(27) = 5.41	0.000010	0.000554	2.025
	Right Precentral Gyrus-Right Intracalcarine Cortex	T(27) = 5.08	0.000024	0.000662	1.902
	Left Lingual Gyrus-Left Postcentral Gyrus	T(27) = 5.15	0.000020	0.000684	1.928
	Left Lingual Gyrus-Right Precentral Gyrus	T(27) = 5.14	0.000021	0.000684	1.924
	Medial Visual Networks (2, −79, 12)-Left Lateral Sensorimotor Networks (−55, −12, 29)	T(27) = 4.96	0.000034	0.000693	1.857
	Medial Visual Networks (2, −79, 12)-.Dorsal Attention Networks (Left Frontal Eye Fields)(−27, −9, 64)	T(27) = 4.86	0.000045	0.000812	1.819
	Right Precentral Gyrus-Right Occipital Pole	T(27) = 4.89	0.000041	0.000962	1.830
	Cluster 2/78	***F(2,26) = 19.83***	***0.000006***	***0.000230***	
	Right Parietal Operculum Cortex-Right Occipital Fusiform Gyrus	T(27) = 6.53	0.000001	0.000086	2.444
	Right Parietal Operculum Cortex-Left Occipital Pole	T(27) = 5.92	0.000003	0.000133	2.216
	Right Parietal Operculum Cortex-Visual Occipital Networks (0, −93, −4)	T(27) = 5.85	0.000003	0.000133	2.190
	Right Parietal Operculum Cortex-Right Occipital Pole	T(27) = 5.83	0.000003	0.000133	2.182
	Right Parietal Operculum Cortex-Left Occipital Fusiform Gyrus	T(27) = 5.33	0.000012	0.000406	1.995
	Right Parietal Operculum Cortex-Left Visual Lateral Networks (−37, −79, 10)	T(27) = 5.06	0.000026	0.000713	1.894
**3**	***Cluster 1/136***	***F(2,26) = 30.56***	***0.000000***	***0.000020***	
	Left Lingual Gyrus-Right Pars Opercularis Inferior Frontal Gyrus	T(27) = 5.83	0.000003	0.000538	2.182
	Right Occipital Fusiform Gyrus-Right Pars Opercularis Inferior Frontal Gyrus	T(27) = 5.65	0.000005	0.000862	2.115
**4**	***Cluster 1/66***	***F(2,26) = 31.16***	***0.000000***	***0.000008***	
	Left Temporal Pole-Right Postcentral Gyrus	T(27) = 5.82	0.000003	0.000557	2.179
**5**	***Cluster 1/210***	***F(2,26) = 29.78***	***0.000000***	***0.000040***	
	Right Posterior Parietal Cortex Frontoparietal Networks (52, −52, 45)-Left Heschl's Gyrus	T(27) = 5.95	0.000002	0.000097	2.227
	Right Posterior Parietal Cortex Frontoparietal Networks (52, −52, 45)-Right Insular Cortex	T(27) = 5.40	0.000011	0.000343	2.021
	Right Parietal Operculum Cortex-Right Posterior Parietal Cortex Frontoparietal	T(27) = 5.72	0.000004	0.000362	2.141

Networks (52, −52, 45)				
Left Parietal Operculum Cortex-Right Posterior Parietal Cortex Frontoparietal Networks (52, −52, 45)	T(27) = 5.80	0.000004	0.000362	2.171
Left Parietal Operculum Cortex-Left Posterior Parietal Cortex Frontoparietal Networks (−46, −58, 49)	T(27) = 5.72	0.000004	0.000362	2.141
Right Posterior Parietal Cortex Frontoparietal Networks (52, −52, 45)-Left Planum Temporale	T(27) = 5.13	0.000022	0.000588	1.920
Left Posterior Parietal Cortex Frontoparietal Networks (−46, −58, 49)-Right Planum Polare	T(27) = 5.48	0.000008	0.000673	2.051
Left Posterior Parietal Cortex Frontoparietal Networks (−46, −58, 49)-Right Parietal Operculum Cortex	T(27) = 5.44	0.000009	0.000673	2.036
Right Angular Gyrus-Right Insular Cortex	T(27) = 5.43	0.000010	0.000778	2.033
Cluster 3/210	***F(2,26) = 24.14***	***0.000001***	***0.000083***	
Right Angular Gyrus-Right Central Opercular Cortex	T(27) = 7.52	0.000000	0.000007	2.815
Right Posterior Parietal Cortex Frontoparietal Networks (52, −52, 45)-Right Heschl’s Gyrus	T(27) = 6.55	0.000001	0.000044	2.452
Right Posterior Parietal Cortex Frontoparietal Networks (52, −52, 45)-Right Planum Temporale	T(27) = 6.43	0.000001	0.000044	2.407
Right Posterior Parietal Cortex Frontoparietal Networks (52, −52, 45)-Right Central Opercular Cortex	T(27) = 6.36	0.000001	0.000044	2.381
Right Planum Temporale-Right Posterior Parietal Cortex Frontoparietal Networks (52, −52, 45)	T(27) = 6.28	0.000001	0.000165	2.351
Leftt Posterior Parietal Cortex Frontoparietal Networks (−46, −58, 49)-Right Central Opercular Cortex	T(27) = 5.34	0.000012	0.000673	1.999
Right Planum Temporale-Right Middle Frontal Gyrus	T(27) = 5.30	0.000013	0.000816	1.984
Right Planum Temporale-Right Angular Gyrus	T(27) = 5.20	0.000018	0.000816	1.947
Right Planum Temporale-Right Lateral Prefrontal Cortex Frontoparietal Networks (41, 38, 30)	T(27) = 5.16	0.000020	0.000816	1.932

	Right Central Opercular Cortex-Right Angular Gyrus	T(27) = 5.35	0.000012	0.000970	2.003
	Cluster 4/210	***F(2,26) = 23.25***	***0.000002***	***0.000085***	
	Right Parietal Operculum Cortex-Right Posterior Division Supramarginal Gyrus	T(27) = 7.05	0.000000	0.000023	2.639
	Cluster 5/210	***F(2,26) = 18.99***	***0.000008***	***0.000346***	
	Right Central Opercular Cortex-Left posterior Superior Temporal Gyrus Language Networks (−57, −47, 15)	T(27) = 6.00	0.000002	0.000347	2.246
**6**	***Cluster 3/66***	***F(2,26) = 18.48***	***0.000010***	***0.000224***	
	Right Precentral Gyrus-Left Insular Cortex	T(27) = 5.85	0.000003	0.000473	2.190
	Right Precentral Gyrus-Right Insular Cortex	T(27) = 5.62	0.000006	0.000473	2.104
**11**	***Cluster 1/120***	***F(2,26) = 30.65***	***0***	***0.000017***	
	Right Inferior Division Lateral Occipital Cortex-Left Posterior Division Middle Temporal Gyrus	T(27) = 5.87	0.000003	0.000488	2.197
	Left Temporooccipital Part Middle Temporal Gyrus-Left Occipital Fusiform Gyrus	T(27) = 5.82	0.000003	0.000561	2.179

Appendix 2. Effect of alpha binaural beats embedded with white noise (AWB) thresholded using a combination of a p-FDR < 0.001 connection-level threshold and a corrected p-FDR < 0.001 cluster-level threshold (MVPA omnibus test).

Circuit	Analysis Unit	Statistic	p-unc	p-FDR	Effect Size (Hedges' g)
**1**	***Cluster 1/78***	***F(2,26) = 51.28***	***0.000000***	***0.000000***	
	Right Precentral Gyrus-Right Supracalcarine Cortex	T(27) = 9.61	0.000000	0.000000	3.597
	Right Precentral Gyrus-Right Cuneal Cortex	T(27) = 8.73	0.000000	0.000000	3.268
	Right Precentral Gyrus-Medial Visual Networks (2, −79, 12)	T(27) = 8.37	0.000000	0.000000	3.133
	Right Precentral Gyrus-Right Lingual Gyrus	T(27) = 7.77	0.000000	0.000001	2.909
	Right Precentral Gyrus-Right Intracalcarine Cortex	T(27) = 7.54	0.000000	0.000001	2.822
	Right Precentral Gyrus-Left Intracalcarine Cortex	T(27) = 7.37	0.000000	0.000002	2.759
	Right Precentral Gyrus-Left Cuneal Cortex	T(27) = 7.25	0.000000	0.000002	2.714
	Superior Sensorimotor Networks (0, −31, 67)-Left Intracalcarine Cortex	T(27) = 7.86	0.000000	0.000003	2.942
	Superior Sensorimotor Networks (0, −31, 67)-Medial Visual Networks (2, −79, 12)	T(27) = 7.50	0.000000	0.000004	2.807

Superior Sensorimotor Networks (0, −31, 67)-Right Lingual Gyrus	T(27) = 6.93	0.000000	0.000010	2.594
Right Precentral Gyrus-Left Supracalcarine Cortex	T(27) = 6.45	0.000001	0.000013	2.414
Right Postcentral Gyrus-Right Supracalcarine Cortex	T(27) = 7.11	0.000000	0.000016	2.661
Right Postcentral Gyrus-Medial Visual Networks (2, −79, 12)	T(27) = 6.92	0.000000	0.000016	2.590
Right Precentral Gyrus-Left Lingual Gyrus	T(27) = 6.30	0.000001	0.000017	2.358
Left Lingual Gyrus-Right Postcentral Gyrus	T(27) = 7.09	0.000000	0.000021	2.654
Superior Sensorimotor Networks (0, −31, 67)-Right Intracalcarine Cortex	T(27) = 6.51	0.000001	0.000023	2.437
Left Lingual Gyrus-Left Postcentral Gyrus	T(27) = 6.70	0.000000	0.000028	2.508
Superior Sensorimotor Networks (0, −31, 67)-Right Supracalcarine Cortex	T(27) = 6.19	0.000001	0.000040	2.317
Superior Sensorimotor Networks (0, −31, 67)-Right Cuneal Cortex	T(27) = 6.14	0.000001	0.000040	2.298
Superior Sensorimotor Networks (0, −31, 67)-Left Cuneal Cortex	T(27) = 5.89	0.000003	0.000066	2.205
Right Postcentral Gyrus-Right Intracalcarine Cortex	T(27) = 5.82	0.000003	0.000127	2.179
Right Postcentral Gyrus-Right Occipital Pole	T(27) = 5.74	0.000004	0.000127	2.149
Right Postcentral Gyrus-Left Intracalcarine Cortex	T(27) = 5.72	0.000004	0.000127	2.141
Right Postcentral Gyrus-Left Cuneal Cortex	T(27) = 5.70	0.000005	0.000127	2.134
Superior Sensorimotor Networks (0, −31, 67)-Right Occipital Fusiform Gyrus	T(27) = 5.50	0.000008	0.000146	2.059
Left Juxtapositional Lobule Cortex (formerly Supplementary Motor Cortex)-Right Occipital Fusiform Gyrus	T(27) = 6.21	0.000001	0.000162	2.325
Left Juxtapositional Lobule Cortex (formerly Supplementary Motor Cortex)-Right Intracalcarine Cortex	T(27) = 6.02	0.000002	0.000162	2.253
Right Precentral Gyrus-Left Occipital Pole	T(27) = 5.41	0.000010	0.000164	2.025
Left Lingual Gyrus-Right Precentral Gyrus	T(27) = 5.70	0.000005	0.000257	2.134
Superior Sensorimotor Networks (0, −31, 67)-Left Lingual Gyrus	T(27) = 5.24	0.000016	0.000258	1.961
Left Juxtapositional Lobule Cortex (formerly Supplementary Motor Cortex)-Left Intracalcarine Cortex	T(27) = 5.64	0.000006	0.000300	2.111
Left Juxtapositional Lobule Cortex (formerly Supplementary Motor Cortex)-Medial Visual Networks (2, −79, 12)	T(27) = 5.52	0.000008	0.000306	2.066
Right Postcentral Gyrus-Right Cuneal Cortex	T(27) = 5.31	0.000013	0.000307	1.988

Right Postcentral Gyrus-Right Lingual Gyrus	T(27) = 5.24	0.000016	0.000328	1.961
Left Lingual Gyrus-Right Lateral Sensorimotor Networks (56, −10, 29)	T(27) = 5.46	0.000009	0.000360	2.044
Medial Visual Networks (2, −79, 12)-Left Postcentral Gyrus	T(27) = 5.81	0.000003	0.000365	2.175
Medial Visual Networks (2, −79, 12)-Left Precentral Gyrus	T(27) = 5.53	0.000007	0.00	2.070
Medial Visual Networks (2, −79, 12)-Right Precentral Gyrus	T(27) = 5.45	0.000009	0.00	2.040
Medial Visual Networks (2, −79, 12)-Right Postcentral Gyrus	T(27) = 5.42	0.000010	0.00	2.029
Medial Visual Networks (2, −79, 12)-Dorsal Attention Networks (Left Intraparietal Sulci) (−39, −43, 52)	T(27) = 5.35	0.00	0.00	2.003
Medial Visual Networks (2, −79, 12)-Left Lateral Sensorimotor Networks (−55, −12, 29)	T(27) = 5.30	0.000013	0.00	1.984
Right Postcentral Gyrus-Left Occipital Fusiform Gyrus	T(27) = 5.11	0.000023	0.00	1.913
Medial Visual Networks (2, −79, 12)-Right Lateral Sensorimotor Networks (56, −10, 29)	T(27) = 5.17	0.000020	0.00	1.935
Connection Superior Sensorimotor Networks (0, −31, 67)-Right Occipital Pole	T(27) = 4.98	0.00	0.00	1.864
Medial Visual Networks (2, −79, 12)-Dorsal Attention Networks (Right Intraparietal Sulci) (39, −42, 54)	T(27) = 5.09	0.00	0.00	1.905
Right Occipital Fusiform Gyrus-Right Juxtapositional Lobule Cortex (formerly Supplementary Motor Cortex)	T(27) = 5.85	0.000003	0.00	2.190
Right Intracalcarine Cortex-Right Postcentral Gyrus	T(27) = 5.85	0.000003	0.00	2.190
Left Occipital Pole-Right Superior Parietal Lobule	T(27) = 5.70	0.000005	0.00	2.134
Left Occipital Pole-Right Postcentral Gyrus	T(27) = 5.58	0.000006	0.00	2.089
Right Postcentral Gyrus-Left Lingual Gyrus	T(27) = 4.94	0.000036	0.00	1.849
Superior Sensorimotor Networks (0, −31, 67)-Left Occipital Fusiform Gyrus	T(27) = 4.83	0.00	0.00	1.808
Left Occipital Pole-Right Precentral Gyrus	T(27) = 5.26	0.000015	0.00	1.969
Left Occipital Pole-Left Postcentral Gyrus	T(27) = 5.20	0.000018	0.00	1.947
Right Intracalcarine Cortex-Right Precentral Gyrus	T(27) = 5.33	0.000013	0.00	1.995
Right Intracalcarine Cortex-Left Postcentral Gyrus	T(27) = 5.29	0.000014	0.00	1.980
Right Intracalcarine Cortex-Left Precentral Gyrus	T(27) = 5.19	0.000018	0.00	1.943

	Left Occipital Pole-Superior Sensorimotor Networks (0, −31, 67)	T(27) = 5.10	0.00	0.00	1.909
	Right Intracalcarine Cortex-Right Lateral Sensorimotor Networks (56, −10, 29)	T(27) = 5.07	0.000025	0.00	1.898
**5**	***Cluster 1/210***	***F(2,26) = 23.95***	***0.00***	***0.00***	
	Right Precentral Gyrus-Right Middle Frontal Gyrus	T(27) = 6.47	0.00	0.00	2.422
	Right Angular Gyrus-Right Lateral Sensorimotor Networks (56, −10, 29)	T(27) = 5.82	0.00	0.00	2.179
	Cluster 2/210	***F(2,26) = 23.26***	***0.00***	***0.00***	
	Right Frontal Pole-Right Lateral Sensorimotor Networks (56, −10, 29)	T(27) = 6.77	0.00	0.00	2.534
	Right Frontal Pole-Left Lateral Sensorimotor Networks (−55, −12, 29)	T(27) = 6.13	0.00	0.00	2.295
	Left Planum Temporale-Right Frontal Pole	T(27) = 6.10	0.00	0.00	2.283
**6**	***Cluster 1/66***	***F(2,26) = 38.11***	***0.000000***	***0.000001***	
	Left Precentral Gyrus-Right Anterior Division Supramarginal Gyrus	T(27) = 6.38	0.000001	0.000126	2.388
	Superior Sensorimotor Networks (0, −31, 67)-Right Anterior Division Supramarginal Gyrus	T(27) = 6.17	0.000001	0.000220	2.310
	Right Lateral SensoriMotor Networks (56, −10, 29)-Left Anterior Division Supramarginal Gyrus	T(27) = 5.97	0.000002	0.000378	2.235
	Right Anterior Division Supramarginal Gyrus-Superior Sensorimotor Networks (0, −31, 67)	T(27) = 5.47	0.000009	0.000699	2.048
	Cluster 2/66	***F(2,26) = 32.14***	***0.000000***	***0.000003***	
	Left Postcentral Gyrus-Right Supramarginal Gyrus Salience Networks (62, −35, 32)	T(27) = 6.07	0.000002	0.000283	2.272
	Left Postcentral Gyrus-Left Rostral Prefrontal Cortex Salience Networks (−32, 45, 27)	T(27) = 5.79	0.000004	0.000300	2.167
	Left Rostral Prefrontal Cortex Salience Networks (−32, 45, 27)-Left Precentral Gyrus	T(27) = 5.95	0.000002	0.000394	2.227
	Left Precentral Gyrus-Right Anterior Insula Salience Networks (47, 14, 0)	T(27) = 5.42	0.000010	0.000810	2.029
	Left Rostral Prefrontal Cortex Salience Networks (−32, 45, 27)-Right Lateral Sensorimotor Networks (56, −10, 29)	T(27) = 5.11	0.000023	0.000923	1.913
	Left Rostral Prefrontal Cortex Salience Networks (−32, 45, 27)-Left Postcentral Gyrus	T(27) = 4.98	0.000032	0.000978	1.864
	Left Rostral Prefrontal Cortex Salience Networks (−32, 45, 27)-Left Lateral	T(27) = 4.94	0.000036	0.000978	1.849

Sensorimotor Networks (−55, −12, 29)				
Cluster 3/66	***F(2,26) = 27.76***	***0.000000***	***0.000007***	
Left Central Opercular Cortex-Left Anterior Division Supramarginal Gyrus	T(27) = 5.87	0.000003	0.000245	2.197
Right Planum Polare-Right Anterior Division Supramarginal Gyrus	T(27) = 5.94	0.000002	0.000407	2.224
Right Parietal Operculum Cortex-Superior Sensorimotor Networks (0, −31, 67)	T(27) = 5.83	0.000003	0.000535	2.182
Right Juxtapositional Lobule Cortex (formerly Supplementary Motor Cortex)-Left Lateral Sensorimotor Networks (−55, −12, 29)	T(27) = 5.67	0.000005	0.000680	2.122
Right Juxtapositional Lobule Cortex (formerly Supplementary Motor Cortex)-Left Precentral Gyrus	T(27) = 5.48	0.000008	0.000680	2.051
Right Anterior Division Supramarginal Gyrus- Right Central Opercular Cortex	T(27) = 5.55	0.000007	0.000699	2.078
Right Central Opercular Cortex-Left Anterior Division Supramarginal Gyrus	T(27) = 5.26	0.000015	0.000834	1.969
Cluster 4/66	***F(2,26) = 27.36***	***0.000000***	***0.000007***	
Left Parietal Operculum Cortex-Dorsal Attention Networks (Right Intraparietal Sulci) (39, −42, 54)	T(27) = 6.53	0.000001	0.000086	2.444
Left Central Opercular Cortex-Dorsal Attention Networks (Left Intraparietal Sulci)(−39, −43, 52)	T(27) = 6.33	0.000001	0.000145	2.370
Dorsal Attention Networks (Left Intraparietal Sulci) (−39, −43, 52)-Left Planum Temporale	T(27) = 5.45	0.000009	0.000517	2.040
Right Central Opercular Cortex-Dorsal Attention Networks (Left Intraparietal Sulci) (−39, −43, 52)	T(27) = 5.54	0.000007	0.000834	2.074
Left Central Opercular Cortex-Left Superior Parietal Lobule	T(27) = 5.25	0.000016	0.000847	1.965
Cluster 7/66	***F(2,26) = 16.31***	***0.000026***	***0.000242***	
Left Rostral Prefrontal Cortex Salience Networks (−32, 45, 27)-Left Juxtapositional Lobule Cortex (formerly Supplementary Motor Cortex)	T(27) = 5.68	0.000005	0.000399	2.126
Left Rostral Prefrontal Cortex Salience Networks (−32, 45, 27)-Right Central Opercular Cortex	T(27) = 5.53	0.000007	0.000399	2.070
Right Central Opercular Cortex-Left Supramarginal Gyrus Salience Networks (−60, −39, 31)	T(27) = 5.32	0.000013	0.000834	1.991

	Cluster 8/66	***F(2,26) = 15.75***	***0.000033***	***0.000272***	
	Dorsal Attention Networks (Left Intraparietal Sulci) (−39, −43, 52)-Right Anterior Insula Salience Networks (47, 14, 0)	T(27) = 5.54	0.000007	0.000517	2.074
	Dorsal Attention Networks (Left Intraparietal Sulci) (−39, −43, 52)-Right Supramarginal Gyrus Salience Networks (62, −35, 32)	T(27) = 5.43	0.000010	0.000517	2.033
**15**	***Cluster 1/171***	***F(2,26) = 22.91***	***0.000002***	***0.000217***	
	Right Inferior Division Lateral Occipital Cortex-Anterior Division Cingulate Gyrus	T(27) = 8.06	0.000000	0.000002	3.017
	Left Inferior Division Lateral Occipital Cortex-Anterior Division Cingulate Gyrus	T(27) = 6.71	0.000000	0.000054	2.512
	Medial Prefrontal Cortex Default Mode Networks (1, 55, −3)-Right Lateral Visual Networks (38, −72, 13)	T(27) = 6.46	0.000001	0.000103	2.418
	Occipital Visual Networks (0, −93, −4)-Anterior Division Cingulate Gyrus	T(27) = 6.14	0.000001	0.000240	2.298
	Right Occipital Pole-Anterior Division Cingulate Gyrus	T(27) = 6.02	0.000002	0.000329	2.253
	Medial Prefrontal Cortex Default Mode Networks (1, 55, −3)-Left Lateral Visual Networks (−37, −79, 10)	T(27) = 5.69	0.000005	0.000390	2.130
	Cluster 2/171	***F(2,26) = 22.02***	***0.000003***	***0.000217***	
	Right Pars Triangularis Inferior Frontal Gyrus- Right Intracalcarine Cortex	T(27) = −6.15	0.000001	0.000233	−2.302
	Right Pars Triangularis Inferior Frontal Gyrus-Left Intracalcarine Cortex	T(27) = −5.76	0.000004	0.000323	−2.156

Appendix 3. Difference of (a) AWN > ABB (b) ABB > AWN thresholded using a combination of a p-uncorrected < 0.05 connection-level threshold and a corrected p-FDR < 0.05.

Group Effect	Circuit	Analysis Unit	Statistic	p-unc	p-FDR	Effect Size (Hedges' g)
**a) Difference AWN > ABB**	**18**	***Cluster 1/210***	***F(2,26) = 12.58***	***0.000151***	***0.031742***	
		Right Cerebelum 10-Subcallosal Cortex	T(27) = 3.55	0.001432	0.116686	1.329
		Right Cerebelum 10-Right Pallidum	T(27) = −3.00	0.005755	0.312688	−1.123
		Vermis 1 2-Right Accumbens	T(27) = −2.38	0.024431	0.332110	−0.891
		Vermis 1 2-Right Caudate	T(27) = −2.22	0.034865	0.332110	−0.831
		Right Accumbens-Right Cerebelum 10	T(27) = −2.71	0.011547	0.496648	−1.014

		Right Accumbens-Right Cerebelum 3	T(27) = −2.68	0.012251	0.496648	−1.003
		Right Accumbens-Vermis 1 2	T(27) = −2.59	0.015235	0.496648	−0.970
		Right Cerebelum 10-Right Accumbens	T(27) = −2.61	0.014525	0.578777	−0.977
		Right Accumbens-Brain-Stem	T(27) = −2.35	0.026215	0.610435	−0.880
		Left Cerebelum 10-Subcallosal Cortex	T(27) = 2.14	0.041176	0.655345	0.801
		Right Pallidum-Right Cerebelum 10	T(27) = −3.09	0.004564	0.720024	−1.157
		Right Pallidum-Left Cerebelum 10	T(27) = −2.82	0.008835	0.720024	−1.056
**b) Difference**	**18**	***Cluster 1/210***	***F(2,26) = 12.58***	***0.000151***	***0.031742***	
**ABB > AWN**		Right Cerebelum 10-Subcallosal Cortex	T(27) = −3.55	0.001432	0.116686	−1.329
		Right Cerebelum 10-Right Pallidum r	T(27) = 3.00	0.005755	0.312688	1.123
		Vermis 1 2-Right Accumbens	T(27) = 2.38	0.024431	0.332110	0.891
		Vermis 1 2-Right Caudate	T(27) = 2.22	0.034865	0.332110	0.831
		Right Accumbens-Right Cerebelum 10	T(27) = 2.71	0.011547	0.496648	1.014
		Right Accumbens- Right Cerebelum 3	T(27) = 2.68	0.012251	0.496648	1.003
		Right Accumbens-Vermis 1 2	T(27) = 2.59	0.015235	0.496648	0.970
		Right Cerebelum 10-Right Accumbens	T(27) = 2.61	0.014525	0.578777	0.977
		Right Accumbens-Brain-Stem	T(27) = 2.35	0.026215	0.610435	0.880
		Left Cerebelum 10-Subcallosal Cortex	T(27) = −2.14	0.041176	0.655345	−0.801
		Right Pallidum-Right Cerebelum 10	T(27) = 3.09	0.004564	0.720024	1.157
		Right Pallidum-Left Cerebelum 10	T(27) = 2.82	0.008835	0.720024	1.056

Appendix 4. Average of AWN and ABB of p-FDR < 0.001 connection-level threshold and a corrected p-FDR < 0.001 cluster-level threshold (MVPA omnibus test).

Circuit	Analysis Unit	Statistic	p-unc	p-FDR	Effect Size (Hedges' g)
1	***Cluster 1/78***	***F(2,26) = 93.60***	***0.000000***	***0.000000***	
	Right Precentral Gyrus-Right Supracalcarine Cortex	T(27) = 11.15	0.000000	0.000000	4.174
	Right Precentral Gyrus-Right Cuneal Cortex	T(27) = 10.14	0.000000	0.000000	3.796

	Right Precentral Gyrus-Medial Visual Networks (2, −79, 12)	T(27) = 10.00	0.000000	0.000000	3.743
	Right Precentral Gyrus- Left Intracalcarine Cortex	T(27) = 8.94	0.000000	0.000000	3.347
	Cluster 2/78	***F(2,26) = 40.32***	***0.000000***	***0.000000***	
	Medial Visual Networks (2, −79, 12)-Right Anterior Division Supramarginal Gyrus	T(27) = 6.13	0.000002	0.000022	2.295
	Right Cuneal Cortex-Left Parietal Operculum Cortex	T(27) = 6.63	0.000000	0.000068	2.482
	Cluster 3/78	***F(2,26) = 25.22***	***0.000001***	***0.000021***	
	Superior Sensorimotor Networks (0, −31, 67)-Left Temporal Occipital Fusiform Cortex	T(27) = 6.96	0.000000	0.000006	2.605
	Right Superior Division Lateral Occipital Cortex-Right Lateral Sensorimotor (56, −10, 29)	T(27) = 5.97	0.000002	0.000369	2.235
**2**	***Cluster 1/91***	***F(2,26) = 22.75***	***0.000002***	***0.000097***	
	Right Supracalcarine Cortex-Left Superior Division Lateral Occipital Cortex	T(27) = 6.13	0.000001	0.000183	2.295
	Medial Visual Networks (2, −79, 12)-Left Superior Division Lateral Occipital Cortex	T(27) = 5.81	0.000003	0.000568	2.175
	Left Intracalcarine Cortex-Left Superior Division Lateral Occipital Cortex	T(27) = 5.01	0.000030	0.000813	1.875
	Cluster 2/91	***F(2,26) = 22.49***	***0.000002***	***0.000097***	
	Right Supracalcarine Cortex-Medial Visual Networks (2, −79, 12)	T(27) = 5.52	0.000007	0.000246	2.066
	Right Supracalcarine Cortex-Right Cuneal Cortex	T(27) = 5.52	0.000008	0.000246	2.066
	Left Supracalcarine Cortex-Medial Visual Networks (2, −79, 12)	T(27) = 5.38	0.000011	0.000363	2.014
	Cluster 3/91	***F(2,26) = 19.71***	***0.000006***	***0.000187***	
	Left Inferior Division Lateral Occipital Cortex-Right Supracalcarine Cortex	T(27) = 6.96	0.000000	0.000029	2.605
	Left Intracalcarine Cortex-Right Occipital Fusiform Gyrus	T(27) = 6.15	0.000001	0.000135	2.302
**3**	***Cluster 1/136***	***F(2,26) = 45.56***	***0.000000***	***0.000000***	
	Left Lingual Gyrus-Right Inferior Frontal Gyrus operculum	T(27) = 6.40	0.000001	0.000121	2.396
	Occipital Visual Networks-Right Lateral Prefrontal Cortex FrontoParietal Networks	T(27) = 5.86	0.000003	0.000168	2.194
	Occipital Visual Networks-Right Middle Frontal Gyrus	T(27) = 5.56	0.000007	0.000243	2.081
	Occipital Visual Networks-Right Angular Gyrus	T(27) = 5.53	0.000007	0.000243	2.070
	Medial Visual Networks-Right Lateral Prefrontal Cortex FrontoParietal Networks	T(27) = 6.08	0.000002	0.000277	2.276
	Left Occipital Fusiform Gyrus-Right Lateral Prefrontal Cortex FrontoParietal	T(27) = 6.07	0.000002	0.000290	2.272

	Networks				
	Occipital Visual Networks-Right Posterior Parietal Cortex FrontoParietal Networks	T(27) = 5.26	0.000015	0.000409	1.969
	Right Occipital Fusiform Gyrus-Right Inferior Frontal Gyrus Operculum	T(27) = 5.66	0.000005	0.000417	2.119
	Right Occipital Fusiform Gyrus-Right Lateral Prefrontal Cortex FrontoParietal	T(27) = 5.59	0.000006	0.000417	2.093
**4**	***Cluster 1/66***	***F(2,26) = 49.37***	***0.000000***	***0.000000***	
	Left Temporal Pole-Right Post Central Gyrus	T(27) = 6.98	0.000000	0.000027	2.613
	Left Temporal Pole-Left Post Central Gyrus	T(27) = 6.03	0.000002	0.000102	2.257
	Left Temporal Pole-Left Parietal Operculum	T(27) = 5.97	0.000002	0.000102	2.235
	Left Temporal Pole-Superior Sensorimotor Networks	T(27) = 5.94	0.000003	0.000102	2.224
	Right Parietal Operculum-Right Temporal Pole	T(27) = 5.73	0.000004	0.000708	2.145
	Right Posterior Middle Temporal Gyrus-Left Lateral SensoriMotor Networks	T(27) = 5.66	0.000005	0.000853	2.119
	Right Parietal Operculum Cortex-Right Posterior Middle Temporal Gyrus	T(27) = 5.36	0.000012	0.000943	2.006
**5**	***Cluster 1/210***	***F(2,26) = 52.81***	***0.000000***	***0.000000***	
	Right PreCentral Gyrus-Right Middle Frontal Gyrus	T(27) = 7.75	0.000000	0.000004	2.901
	Right Angular Gyrus-Right Inferior Colliculus	T(27) = 7.37	0.000000	0.000005	2.759
	Right Angular Gyrus-Right Lateral Sensorimotor Networks	T(27) = 7.16	0.000000	0.000006	2.680
	Right Lateral PreFrontal Cortex FrontoParietal Networks-Right lateral Sensorimotor Networks	T(27) = 6.79	0.000000	0.000022	2.542
	Left Planum Temporal-Right Lateral PreFrontal Cortex FrontoParietal Networks	T(27) = 6.71	0.000000	0.000027	2.512
**6**	***Cluster 1/66***	***F(2,26) = 46.33***	***0.000000***	***0.000000***	
	Right Planum Temporal-Dorsal Attention Networks (Right Intraparietal Sulci)	T(27) = 6.38	0.000001	0.000064	2.388
	Cluster 2/66	***F(2,26) = 45.51***	***0.000000***	***0.000000***	
	Right Planum Parietale-Right Anterior Supramarginal Gyrus	T(27) = 7.96	0.000000	0.000002	2.980
	Right CO-Left Anterior Supramarginal Gyrus	T(27) = 6.56	0.000000	0.000043	2.456
	Cluster 3/66	***F(2,26) = 44.91***	***0.000000***	***0.000000***	
	Left Lateral Sensorimotor Networks-Right Anterior Supramarginal Gyrus	T(27) = 6.76	0.000000	0.000035	2.530
	Cluster 4/66	***F(2,26) = 44.33***	***0.000000***	***0.000000***	
	Left Parietal Operculum Cortex-Dorsal Attention Networks (Right Intraparietal Sulci)	T(27) = 7.58	0.000000	0.000006	2.837

	Cluster 5/66	**F(2,26) = 42.04**	**0.000000**	**0.000000**	
	Left Rostral Prefrontal Cortex Salience Networks-Left PreCentral Gyrus	T(27) = 6.90	0.000000	0.000011	2.583
**7**	***Cluster 3/231***	***F(2,26) = 28.70***	***0.000000***	***0.000019***	
	Left Lateral Prefrontal Cortex FrontoParietal Networks-Right Superior Frontal Gyrus	T(27) = 5.94	0.000003	0.000136	2.224
	Cluster 4/231	***F(2,26) = 28.04***	***0.000000***	***0.000019***	
	Left Lateral Prefrontal Cortex FrontoParietal Networks-Right Paracingulate Gyrus	T(27) = 6.34	0.000001	0.000070	2.373
	Cluster 5/231	***F(2,26) = 26.66***	***0.000001***	***0.000023***	
	Right Inferior Frontal Gyrus Operculum -Left Posterior Middle Temporal Gyrus	T(27) = 6.27	0.000001	0.000168	2.347
**8**	***Cluster 1/55***	***F(2,26) = 31.72***	***0.000000***	***0.000006***	
	Right Subgenual Cingulate Cortex-Left Posterior Superior Temporal Gyrus	T(27) = 6.05	0.000002	0.000302	2.265
	Cluster 3/55	***F(2,26) = 24.02***	***0.000001***	***0.000018***	
	Medial Visual Networks-Right Anterior Insula Salience Networks	T(27) = 5.60	0.000006	0.000320	2.096
**9**	***Cluster 3/136***	***F(2,26) = 17.20***	***0.000017***	***0.000770***	
	Anterior Cerebellar Networks-Right PreCentral Gyrus	T(27) = 6.28	0.000001	0.000166	2.351
	Cluster 5/136	***F(2,26) = 15.63***	***0.000035***	***0.000948***	
	Right Cerebelum 1-Left Subgenual Cingulate Cortex	T(27) = 6.10	0.000002	0.000268	2.283
**10**	***Cluster 2/105***	***F(2,26) = 24.90***	***0.000001***	***0.000048***	
	Left Middle Frontal Gyrus-Dorsal Attention Networks (Left Intraparietal Sulci)	T(27) = 5.76	0.000004	0.000215	2.156
	Cluster 4/105	***F(2,26) = 17.68***	***0.000014***	***0.000373***	
	Left Middle Frontal Gyrus-Right Lateral Visual Networks	T(27) = 6.25	0.000001	0.000089	2.340
**11**	***Cluster 1/120***	***F(2,26) = 50.59***	***0.000000***	***0.000000***	
	Right Inferior Division Lateral Occipital Cortex-Left Posterior Middle Temporal Gyrus	T(27) = 6.95	0.000000	0.000030	2.602
	Left Posterior Middle Temporal Gyrus-Right Occipital Pole	T(27) = 6.62	0.000000	0.000069	2.478
	Right Inferior Division Lateral Occipital Cortex-Left Temporooccipital Middle Temporal Gyrus	T(27) = 5.97	0.000002	0.000189	2.235
	Left Posterior Middle Temporal Gyrus-Occipital Visual Networks	T(27) = 5.66	0.000005	0.000419	2.119
	Left Anterior Inferior Temporal Gyrus-Right Occipital Fusiform Gyrus	T(27) = 5.85	0.000003	0.000509	2.190

	Cluster 3/120	***F(2,26) = 24.96***	***0.000001***	***0.000036***	
	Right Posterior Middle Temporal Gyrus-Left Occipital Pole	T(27) = 6.31	0.000001	0.000152	2.362
	Right Posterior Middle Temporal Gyrus-Occipital Visual Networks	T(27) = 5.64	0.000005	0.000282	2.111
	Right Posterior Middle Temporal Gyrus-Right Occipital Pole	T(27) = 5.55	0.000007	0.000282	2.078
**12**	***Cluster 1/105***	***F(2,26) = 22.77***	***0.000002***	***0.000203***	
	Medial Prefrontal Cortex Default Mode Networks-Left Inferior Colliculus	T(27) = 6.02	0.000002	0.000329	2.253
**13**	***Cluster 4/105***	***F(2,26) = 18.74***	***0.000009***	***0.000239***	
	Anterior Cingulate Cortex Salience Networks-Right Temporal Occipital Fusiform Cortex	T(27) = 6.15	0.000001	0.000230	2.302
**14**	***Cluster 4/171***	***F(2,26) = 17.39***	***0.000016***	***0.000685***	
	Left Posterior Temporal Fusiform Cortex-Left Lateral Sensorimotor Networks	T(27) = 6.08	0.000002	0.000283	2.276
**15**	***Cluster 1/171***	***F(2,26) = 34.09***	***0.000000***	***0.000009***	
	Right Inferior Division Lateral Occipital Cortex-Anterior Division Cingulate Gyrus	T(27) = 7.42	0.000000	0.000009	2.778
	Medial Prefrontal Cortex Default Mode Networks-Right Laretal Visual Networks	T(27) = 7.40	0.000000	0.000009	2.770
	Left Inferior Division Lateral Occipital Cortex-Anterior Division Cingulate Gyrus	T(27) = 7.24	0.000000	0.000014	2.710
	Occipital Visual Networks-Anterior Division Cingulate Gyrus	T(27) = 7.19	0.000000	0.000016	2.691
**16**	***Cluster 3/253***	***F(2,26) = 18.39***	***0.000011***	***0.000645***	
	Right Lingual Gyrus-Left Middle Frontal Gyrus	T(27) = 5.60	0.000006	0.000944	2.096
	Right Lingual Gyrus-Left Superior Frontal Gyrus	T(27) = 5.36	0.000012	0.000944	2.006
**17**	***Cluster 1/253***	***F(2,26) = 43.98***	***0.000000***	***0.000001***	
	Right Cerebellum 8-Right temporooccipital Inferior Temporal Gyrus	T(27) = 6.45	0.000001	0.000107	2.414
	Left Lateral Visual Networks-Right Cerebellum 8	T(27) = 5.60	0.000006	0.000997	2.096
	Cluster 2/253	***F(2,26) = 37.43***	***0.000000***	***0.000003***	
	Left Cerebellum 6-Right Inferior Division Lateral Occipital Cortex	T(27) = 6.28	0.000001	0.000132	2.351
	Left Posterior Temporal Fusiform Cortex-Right Superior Lateral Occipital Cortex	T(27) = 5.72	0.000004	0.000362	2.141
	Cluster 4/253	***F(2,26) = 20.44***	***0.000005***	***0.000255***	
	Left Posterior Temporal Fusiform Cortex-Right Occipital Pole	T(27) = 6.05	0.000002	0.000304	2.265
	Cluster 6/253	***F(2,26) = 17.01***	***0.000019***	***0.000799***	

	Left Cerebellum 6-Left Temporal Occipital Fusiform Cortex	T(27) = 5.13	0.000021	0.000701	1.920
**20**	***Cluster 3/78***	***F(2,26) = 17.15***	***0.000018***	***0.000462***	
	Precuneous -Right Frontal Pole	T(27) = 5.33	0.000013	0.000878	1.995
	Cluster 4/78	***F(2,26) = 15.63***	***0.000035***	***0.000681***	
	Precuneous -Right Lateral Prefrontal Cortex FrontoParietal Networks	T(27) = 5.89	0.000003	0.000464	2.205
	Precuneous -Right Angular Gyrus	T(27) = 5.24	0.000016	0.000878	1.961

AIMS Press

© 2025 the Author(s), licensee AIMS Press. This is an open access article distributed under the terms of the Creative Commons Attribution License (https://creativecommons.org/licenses/by/4.0)
